# Supplementary material for: Highly contractile 3D tissue engineered skeletal muscles from human iPSCs reveal similarities with primary myoblast-derived tissues
Source: Stem Cell Reports. 2023 Sep 28;18(10):1954–71. doi: 10.1016/j.stemcr.2023.08.014 (PMC10656354; doi:10.1016/j.stemcr.2023.08.014)
Supplement: Document S1. Figures S1–S7 and Tables S1 and S2 [file mmc1.pdf]

Stem Cell Reports, Volume *18*

Supplemental Information

Highly contractile 3D tissue engineered skeletal muscles from human iPSCs reveal similarities with primary myoblast-derived tissues

Erik van der Wal, Alessandro Iuliano, Stijn L.M. in 't Groen, Anjali P. Bholasing, Dominik Priesmann, Preeti Sharma, Bianca den Hamer, Vittorio Saggiomo, Marcus Krüger, W.W.M. Pim Pijnappel, and Jessica C. de Greef

Supplemental figures

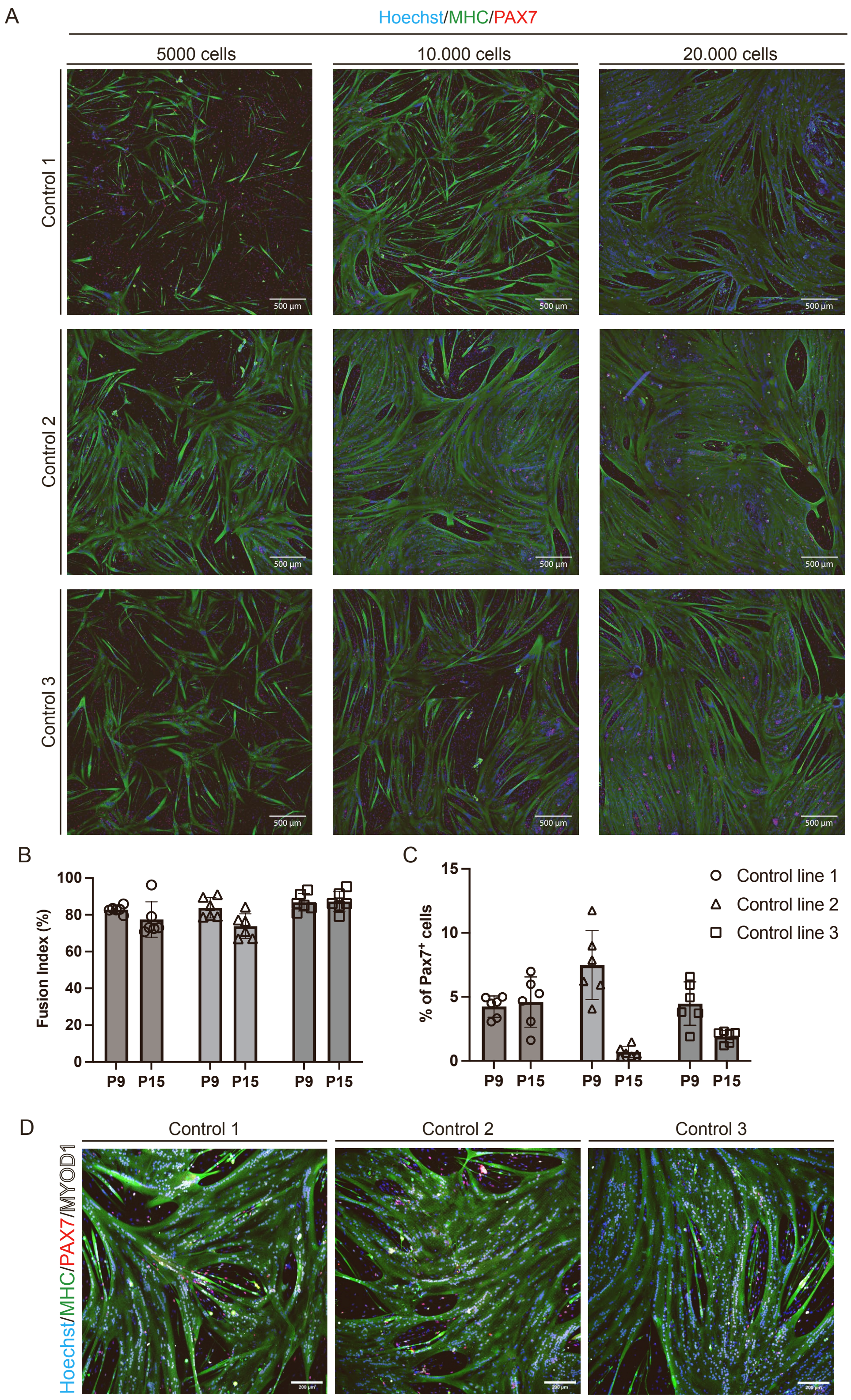

Figure S1 (related to Figure 1). Control 1-3 myogenic progenitors. (A) Staining of MPs of control 1-3 differentiated for 4 days with MYH (green) and PAX7 (red) at different cell densities. Nuclei were

stained with Hoechst (blue). (B) Quantification of fusion index for control 1-3 at passage 9 (P9) and passage 15 (P15) of culture. C) Quantification of percentage of Pax7$^+$ cells in the culture of differentiated MPs of control 1-3 at P9 and P15. D) Immunostaining of MPs from control lines 1-3 differentiated for 4 days with MYH (green), PAX7 (red), MYOD1 (white) and nuclear staining with Hoechst (blue).

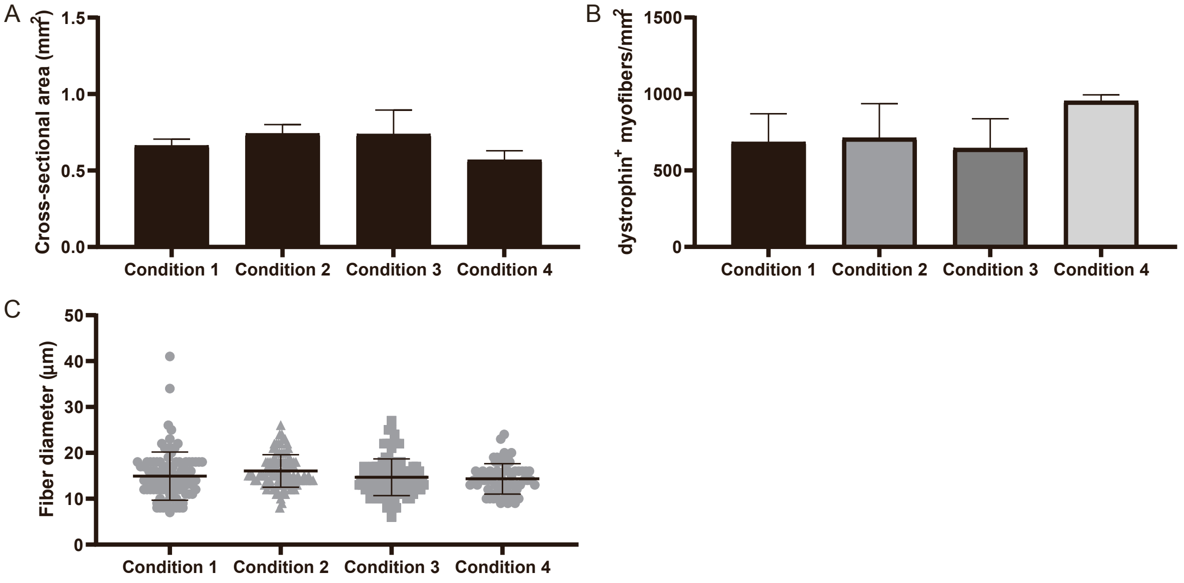

Figure S2 (related to Figure 2). Cross-sectional area, number of dystrophin$^+$ myofibers and myofiber diameter. A) 3D-TESMs were differentiated in conditions 1-4 and on day 7 of differentiation the cross-sectional area was determined. B) Same as (A), but with the number of dystrophin$^+$ myofibers corrected for the cross-sectional area (see Figure 2C). C) Same as (B), but then for myofiber diameter. Five random fields were selected per cross-section and at least 100 myofibers were measured. Data is derived from three independent 3D-TESMs and expressed as mean ± SD.

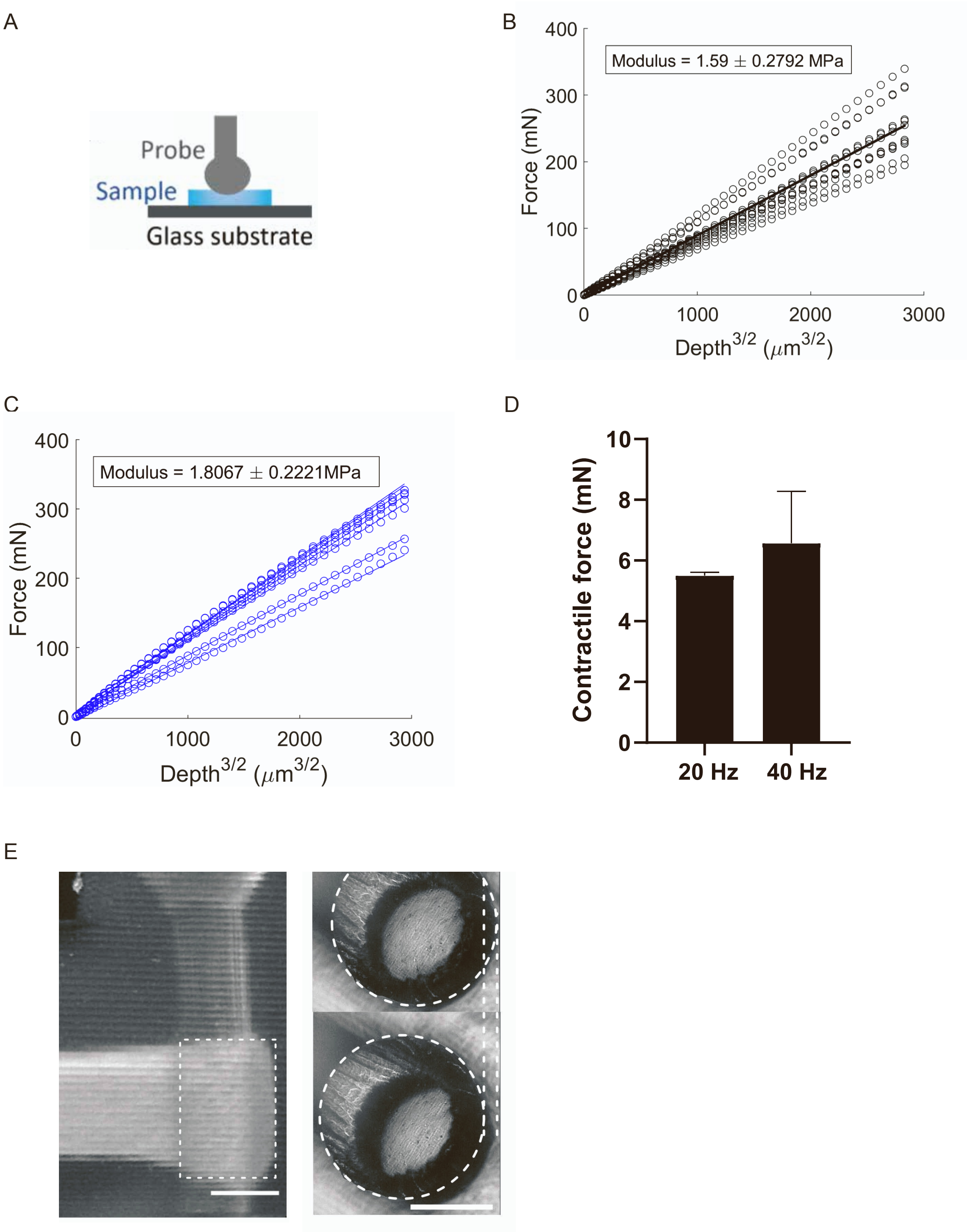

Figure S3 (related to Figures 3 and 5). PDMS stiffness and absolute contractile force. A) PDMS stiffness was measured using indentation method. B) Measured Young's Modulus for the Direct Peeling platform from 15 independent PDMS chambers. C) Measured Young's Modulus of Ecoflex Replica platform from 7 independent PDMS chambers. Plotted line indicates average of all samples. D) Absolute contractile force of 3D-TESMs from control 1 myogenic progenitors stimulated with 20 Hz and 40 Hz on day 7 of differentiation from three independent 3D-TESMs Data is expressed as mean ± SD. E) Position of 3D-TESMs on the pillar of an Ecoflex chamber (left) and top view of pillar displacement (right) before and during stimulation.

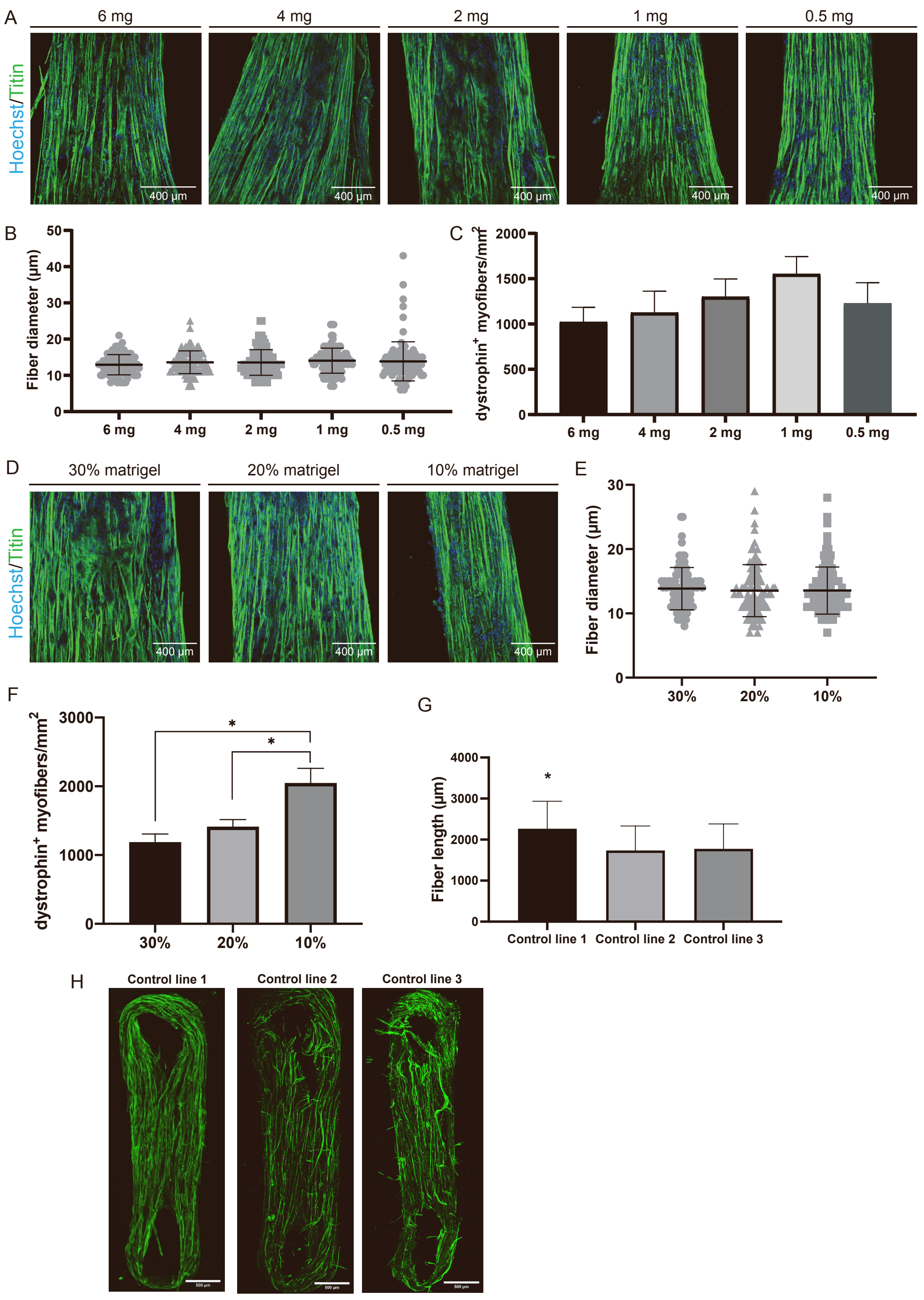

Figure S4 (related to Figure 4). Varying fibrinogen and Matrigel concentrations in the hydrogel of 3D-TESMs. A) 3D-TESMs stained for titin (green) and Hoechst (blue) using whole mount staining on day 7 of differentiation. B) Average myofiber diameter ± SD measured from 3 random fields on 3 cross-sections per fibrinogen concentration derived from Figure 4A. C) Same as (B) but for the average

number of dystrophin+ myofibers. D) Same as (A) but for different Matrigel concentrations. E) Same as (B) showing myofiber diameter quantified from cross-sections of Figure 4E. F) Same as (E) but for the average number of dystrophin+ myofibers**.** Data is derived from three independent 3D-TESMs and expressed as mean ± SD. G) Quantification of fiber length (n=20) in 3D-TESMs generated with the Ecoflex Replica platform from the three control lines. H) Whole-tissue immunostaining for titin (green) of representative tissues generated in the Ecoflex Replica platform for control lines 1-3.

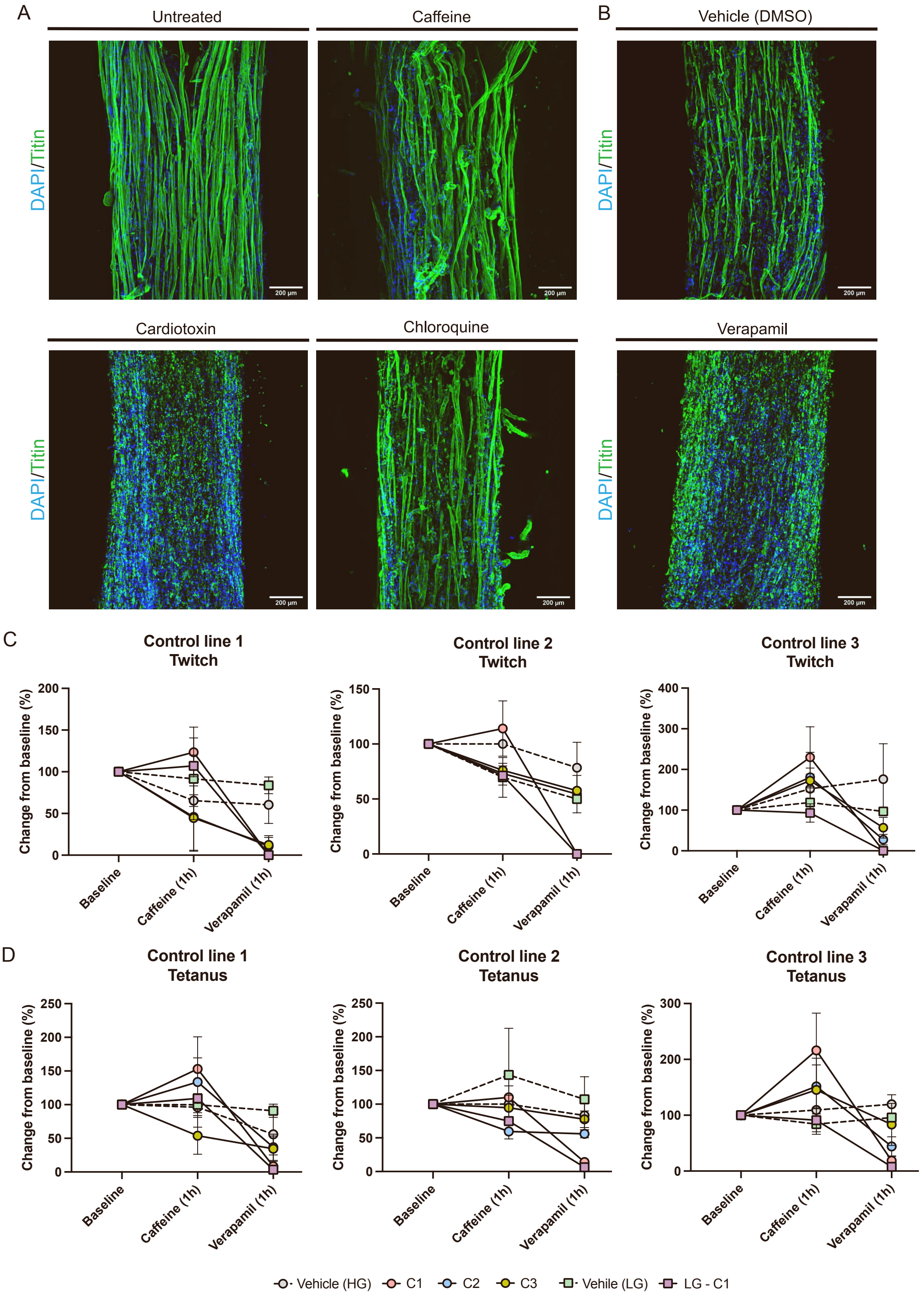

Figure S5 (related to Figure 5). Drug testing on 3D-TESMs from Ecoflex Replica platform. A) Whole mount staining of 3D-TESMs treated with drugs (caffeine, cardiotoxin, chloroquine) for 6 hours using an antibody against titin (green) and nuclei stained with DAPI (blue). C) Same as (D) but then for verapamil, incubated for 1 hour. C) Quantification of change in absolute twitch force after 1h of

administration of caffeine and verapamil, for control lines 1-3. Dashed lines identify vehicle controls. D) Same as (C), but for absolute tetanic force.

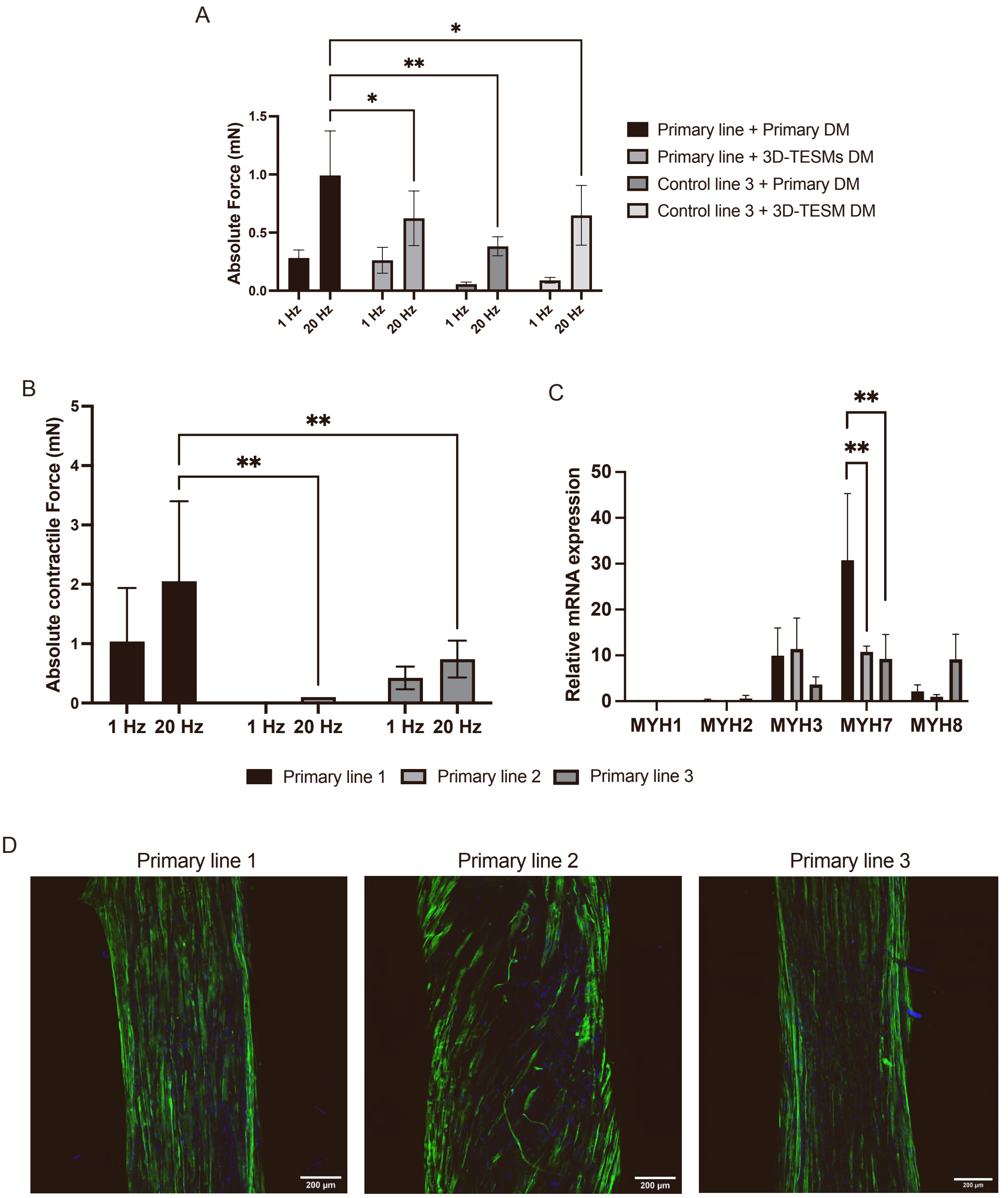

Figure S6 (related to Figure 6). Comparison of three independent primary myoblast lines. A) Comparison of contractile force generated by primary myoblasts derived tissues and myogenic progenitors-derived 3D-TESMs under different media formulations. B) Absolute force quantification for primary lines 1, 2 and 3 at D7 of differentiation of 3D-TESMs generated from the Ecoflex Replica platform. C) RT-qPCR gene expression analysis for the three primary lines. D) Whole-mount immunostaining of representative tissues of the three primary lines. Important disclaimer: any difference in the gene expression data relative to the primary lines compared to the main manuscript is due to intrinsic operator-dependent variability.

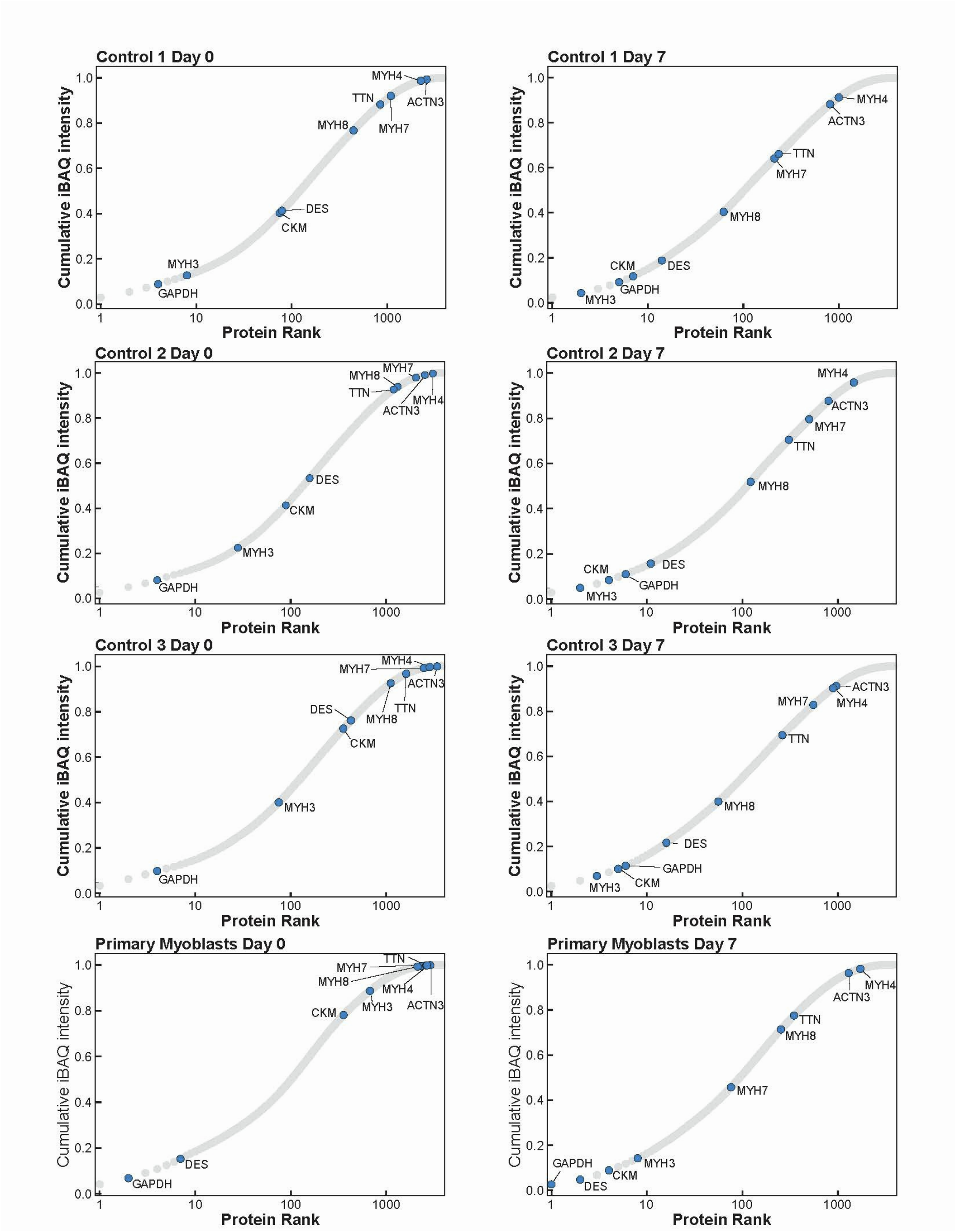

Figure S7 (related to Figure 7). Protein rankings in hiPSC-derived and primary myoblast-derived 3D-TESMs. Protein ranking overview at day 0 (left) and day 7 (right) of differentiation for 3D-TESMs derived from controls 1-3 and primary myoblasts. Protein ranking is based on iBAQ of individual proteins on the x-axis; whereby rank 1 represents the most abundant protein in the sample, while the cumulative iBAQ intensity is represented on the y-axis. Data is derived from three independent 3D-TESMs and expressed as mean.

Supplemental methods

Fabrication of Direct Peeling and Ecoflex Replica-based PDMS chambers

PDMS chambers for the Direct Peeling platform (50 µl) or Ecoflex Replica platform (15 µl) were fabricated as previously described (Iuliano et al., 2020). Briefly, for the Direct Peeling platform, negative molds were printed with an Ultimaker 2^{+} (Ultimaker) FDM printer with ABS material (Ultimaker). SYLGARD™ 184 (PDMS) Base and Curing agent (Dow Corning) were thoroughly mixed in a 10:1 ratio, degassed under vacuum, poured on top of the negative Direct Peeling mold and incubated for 2 hours at 75 °C. For the Ecoflex Replica platform, a two-step replica molding methodology was performed. Positive master molds were generated with SLA 3D printing (Form 3, Formlabs) and negative molds were generated of Ecoflex-00-30 material (Smooth-On Inc). PDMS chambers were released after casting by stretching of the Ecoflex-00-30 negative mold. Prior to generation of 3D-TESMs, PDMS chambers were pretreated with 1% Pluronic F127 (Sigma-Aldrich) for at least 1 hour at room temperature.

Human primary myoblast culture

Human primary myoblasts (Rf745.1) received from the University of Rochester biorepository (https://www.urmc.rochester.edu/neurology/fshd-center/research-info/sharingbiologicalresources.aspx) (Balog et al., 2012) were cultured using previously defined conditions (Madden et al., 2015). In short, during expansion myoblasts were cultured on ECM-coated dishes in DMEM low glucose supplemented with SkGM bullet kit (Lonza: CC-3245) excluding insulin (GM primary). For 3D-TESM formation of human primary myoblasts, the same hydrogel composition and number of cells were used as for 3D-TESMs generated with myogenic progenitors. After formation, 3D-TESMs were first cultured in GM primary medium supplemented with 1.5 mg/ml 6-ACA and after 2 days differentiation was induced with DM primary medium (DMEM low glucose supplemented with 2% horse serum (Gibco), 2 mg/ml 6-ACA and 10 µg/ml insulin).

RNA isolation

Prior to RNA isolation, 3D-TESMs were washed once with PBS, detached from pillars, snap-frozen in liquid nitrogen and stored at -80 °C. For RNA isolation 3D-TESMs were dissolved in 700 µl QIAzol lysis buffer (Qiagen) and disrupted with a tissueRuptor (Qiagen) for 5 seconds. After disruption, RNA was isolated with the mRNeasy mini kit or RNeasy micro kit (Qiagen) including the DNase step, all according to the manufacturer’s manual.

cDNA synthesis and RT-qPCR

Equal amounts of RNA were used for cDNA synthesis using Fermentas cDNA synthesis kit (Fermentas) according to the manufacturer's manual. For RT-qPCR, cDNA was either 5× or

10× diluted and performed as described previously (van der Wal et al., 2017). Primers are shown in Table S1.

Whole mount immunofluorescence staining

3D-TESMs were washed once with PBS, fixed with 4% paraformaldehyde (PFA; Sigma-Aldrich) for 1 hour at room temperature and stored in PBS at 4 °C. For staining, 3D-TESMS were blocked for 1 hour in blocking buffer containing 3% BSA, 0.1% Tween-20 and 0.3% Triton X-100 (all Sigma-Aldrich). Primary and secondary antibodies (Table S1) were diluted in antibody dilution buffer (0.1% BSA, 0.1% Tween-20, 0.3% Triton-X100). Primary antibodies were incubated for 1 hour at room temperature while shaking (150 rpm), followed by washing with PBS-0.1% Tween-20 and with PBS. Thereafter, 3D-TESMs were incubated for 30 minutes at room temperature with secondary antibodies combined with Hoechst nuclear staining. 3D-TESMs were washed once with PBS-0.1% Tween-20 and imaged using an Andor Dragonfly 200 or Leica TCS SP5 Confocal microscope.

Indentation method to measure Young's Modulus of PDMS chambers

Young's modulus of the samples was measured with a custom-made indentation set-up. A spherical glass probe with a diameter of 2 mm was used to indent PDMS sample, which consisted of a flat piece cut from the chamber and was fixed onto a glass substrate via plasma bonding. Step size was 5 µm with an indentation depth of 200 µm. The elastic response was measured via a load cell (250 gm, Futek) with a sampling rate of 1000. An average of 1000 points was plotted versus the indentation depth. Multiple experiments were performed on each sample with varying locations. Young's Modulus (Y) was determined by fitting the indentation depth (*d*), as a function of measured indenter load (F) during approach via following equation (Harding and Sneddon, 1945):

$$F = \frac{4}{3}\frac{Y}{1-\nu^2}d^{3/2}R^{1/2}$$

R is the radius of the indenting sphere (1 mm) and n is the Poisson ratio (0.5). The slope of the linear fit allowed us to extract Young's modulus (Y).

Drug treatment of 3D-TESMs

For drug treatments, 3D-TESMs were incubated with either caffeine (C0750-100G, Sigma-Aldrich), chloroquine (C6628-25G, Sigma-Aldrich-) or cardiotoxin from *Naja pallida* (L8102, Latoxan), all dissolved in water. Verapamil (V4629-1G, Sigma-Aldrich) was dissolved in DMSO as per manufacturer instructions, DMSO was included as a vehicle treatment at a final concentration of 0.1 % v/v. All drugs were diluted in differentiation medium. 3D-TESMs treated with caffeine, chloroquine and cardiotoxin were incubated for 6 hours before analysis. 3D-TESMs treated with verapamil and vehicle were incubated for 1 hour before analysis.

Proteomic analysis

3D-TESMs generated in Ecoflex Replica-based chambers were used for proteomic analysis. For analyses, three 3D-TESMs per biological replica were pooled. Three biological replicas were used per cell line and differentiated for 0 or 7 days. After culturing, all 3D-TESMs were immediately snap-frozen in liquid nitrogen and stored at -80 °C. As a negative control, three cell-free tissues were included containing only fibrinogen, thrombin and Matrigel and were snap-frozen after 30 minutes of polymerization. Tissues were resuspended in 50 µL urea buffer (6 M urea, 2 M thiourea in 10 mM HEPES/KOH pH 8.0) and sonicated for complete disruption. Proteins were reduced and alkylated with 5 mM TCEP and 15 mM CAA at room temperature for 30 minutes. Samples were digested with endoproteinase Lys-C (Wako) at an enzyme_substrate ratio of 1:100 for 3 hours, diluted with three sample volumes of 50 mM ammonium bicarbonate (ABC) buffer and further digested with Trypsin (Sigma Aldrich) at an enzyme_substrate ratio of 1:100 at room temperature overnight. Proteases were inactivated by adding formic acid to 1% and undigested proteins were removed by centrifugation (20,000 x g, RT, 10 min). Peptides were desalted on SDB-RPS StageTips.

Proteomics data were acquired on an Orbitrap Eclipse Tribrid mass spectrometer equipped with a FAIMS PRO device, coupled to an EASY-nLC 1200 UHPLC system (Thermo Scientific) equipped with a 50 cm column packed in-house with C18 *Poroshell* beads (2.7 µm, Agilent). A two-hour gradient within a binary solvent system consisting of 0.1% formic acid (buffer A) and 0.1% formic acid in 80% acetonitrile (buffer B) was used. Buffer B was increased from 4% to 35% within 98 minutes and further to 55% within 12 minutes. The gradient was ramped up to 95% buffer B within 4 minutes and maintained for 6 minutes to elute any remaining peptides. Full MS scans from 350-1400 m/z were acquired at a resolution of 60,000, an AGC target of 1.2E7 and a maximum injection time of 50 ms. Fragmentation scans were acquired at a resolution of 15,000, an AGC target of 1.5E6 and a maximum injection time of 22 ms. A total of 12, 10 and 8 fragmentation scans were acquired at FAIMS compensation voltages of -50 V, -65 V and -80 V, respectively.

Raw files were processed in MaxQuant (v. 1.6.14) using implemented Andromeda search engine (Cox et al., 2011). Peptides and proteins were identified using human Uniprot database (FASTA file downloaded 13/9/2019) with common contaminants. All parameters were set to default values and Trypsin/P was chosen as protease. Methionine oxidation and N-terminal acetylation were set as variable modifications and cysteine carbamidomethylation was considered a fixed modification. The “match between runs” algorithm was enabled, and label-free quantification (LFQ) was performed based on proteotypic peptide intensities.

Statistical analysis was performed in Perseus (v. 1.6.15) (Tyanova et al., 2016). Potential contaminants and reverse peptides were filtered and LFQ intensities were log2-transformed. Data were normalized by column-wise median subtraction and two-sided t-tests were performed to identify differentially expressed proteins. Permutation-based false discovery rate (FDR) estimation was performed with a Fudge factor (S_0) of 0.1 and 500 permutations.

The FDR cutoff was set to 0.05. Identified protein groups were annotated with Gene Ontology terms for Cellular Component, Molecular Function and Biological Process. Graphical visualization was done in Instant Clue.

Tissue sectioning

For the Direct Peeling-based chambers, detached 3D-TESMs were fixed for 1 hour with 4% PFA, washed with PBS and incubated for 2 days in 30% sucrose solution (Sigma-Aldrich) in PBS at 4 °C. Next, 3D-TESMs were embedded in OCT in plastic cryomolds (both Tissue-Tek), snap frozen in liquid nitrogen cooled isopentane (Sigma-Aldrich) and stored at -80 °C. 20 µM thick sections were generated and after cryosectioning slices were stored at -20 °C. For staining, antigen retrieval was performed with Tris/EDTA (10 mM Tris, 1.25 mM EDTA pH9) buffer for 15 minutes while boiling. Staining was performed as described above and after the last step, tissues were dried and mounted with Prolong Diamond antifade mountant (Thermo Fisher Scientific).

Due to the small size of 3D-TESMs generated in Ecoflex Replica-based chambers, either gelatin embedding or paraffin embedding was performed. Gelatin embedding is gentler on tissues, thus it was used to maintain shape and size of engineered tissues for later quantifications. Before embedding, 3D-TESMS were fixed with 4% PFA and then incubated in 10% sucrose dissolved in water overnight at 4 °C. Multiple tissue replicas were then placed on a single block of gelatin containing 30% sucrose and covered with an additional layer of the same solution for a complete embedding. Subsequently, embedded tissue blocks were incubated overnight at 4 °C in a 30% sucrose water-based solution for equilibration and ultimately subjected to a final fixation step in the same solution with addition of 10% PFA ON at 4 °C. Tissue blocks were ultimately freeze-cut in sections of 30 µm thickness using a cryotome. Before mounting on glass slides for imaging, sections were subjected to a free-floating immunostaining procedure identical to whole mount immunostaining used for entire tissues. Paraffin embedding was instead chosen merely for fast qualitative assessments. Fixed tissues were embedded in paraffin blocks through a standard protocol using a histological sample preparation machine (EFTP, Intelsint, Italy) and cut in sections of 6 µm thickness using a microtome. Sections were then mounted on glass slides and subjected to immunostaining.

Image analysis, quantification of cross-sectional area (CSA), myofiber diameter and length

To process the images obtained after immunofluorescence staining the software ImageJ was used. The same software was used to perform quantification of CSA and fiber diameter and length from the images of the 3D-TESMs. Three (n=3) tissues per line and per condition were used for each analysis, cross sections belonging to the central portion of each tissue were chosen for further quantifications. Two different levels of image threshold were chosen to analyze the CSA and the fiber diameter, respectively. The levels of threshold were maintained constant across multiple pictures. To quantify the fiber diameter, we chose only fibers that were both positive for dystrophin immunostaining and showed a complete positive

perimeter. All the candidate fibers visible in each section were selected. In order to minimize the possible bias coming from the irregular shape of the fibers, we semi-randomly selected the axis of each fiber to be chosen as diameter. To quantify the length of the fibers we performed tile scans of whole-mount immunostained tissues. Three tissues per line were chosen and the length of fibers positive for titin was measured. Only fibers whose beginning and end was clearly trackable were chosen and segmented lines were traced along each fiber (n=20 per tissue).

Data and materials availability

All data needed to evaluate conclusions in the paper are present in the paper and/or the supplemental information. Raw/processed data required to reproduce these findings cannot be shared at this time due to technical or time limitations. Additional data related to this paper may be requested from the authors.

Supplemental tables

Table S1. Antibodies and primers used

Name	Dilution or Sequence 5'-3'	Company	Assay
Mouse-anti-MF20	1:50	DSHB	IF
Mouse-anti-PAX7	1:100	DSHD	IF
Mouse-anti-Titin	1:50	DSHB	IF, IHC
Mouse-anti-Brachyury	1:200	R&D (AF2085)	IF
Rabbit-anti-Dystrophin	1:100 or 1:500	Abcam (ab15277)	IHC
MYH1_fw	CTCCTCTTTGTTGGGGCAAC	IDTDNA	RT-qPCR
MYH1_rv	CAGCTTATTCAAATTCTCCC	IDTDNA	RT-qPCR
MYH2_fw	TAAAAAGCTCCAAGAACTGT	IDTDNA	RT-qPCR
MYH2_rv	TGCGCTCCCTTTCAGACTTT	IDTDNA	RT-qPCR
MYH3_fw	CTTGTGGGCGGAGGTCTG	IDTDNA	RT-qPCR
MYH3_rv	AGCAGCTATGCCGAACACTT	IDTDNA	RT-qPCR
MYH4_fw	CTTGAAGTAGTTGTCTGCTTTG	IDTDNA	RT-qPCR
MYH4_rv	GGCTGCAGGTTATTGATGGC	IDTDNA	RT-qPCR
MYH7_fw	CTGTCCAAGTTCCGCAAGGT	IDTDNA	RT-qPCR
MYH7_rv	TCATTCAAGCCCTTCGTGCC	IDTDNA	RT-qPCR
MYH8_fw	ATTTCCACCAAGAACCCA	IDTDNA	RT-qPCR
MYH8_rv	AAAGGATTCTGCCTCTGG	IDTDNA	RT-qPCR

Table S2. Medium composition of condition 1-4

Condition	Composition	References
Condition 1	DMEM High glucose, 1% penicillin, 1% ITS-X, 2 mg/ml 6-ACA	(Borchin et al., 2013)
Condition 2	DMEM Low glucose, 1% penicillin, 2% N2, 2 mg/ml 6-ACA	(Rao et al., 2018)

Condition 3	DMEM Low glucose, 2% horse serum, 10 µg/ml insulin, 1% penicillin, 2 mg/ml 6-ACA	(Afshar et al., 2020; Afshar Bakooshli et al., 2019)
Condition 4	DMEM High glucose, 1% Pen, 1% ITS-X, 1% KOSR, 2 mg/ml 6-ACA	(van der Wal et al., 2018)

Supplemental references

Balog J., Thijssen PE., de Greef JC., Shah B., van Engelen BG., Yokomori K., Tapscott SJ., Tawil R., van der Maarel SM. (2012) Correlation analysis of clinical parameters with epigenetic modifications in the DUX4 promoter in FSHD. Epigenetics. *6*, 579-84.

Cox, J., Neuhauser, N., Michalski, A., Scheltema, R.A., Olsen, J.V., and Mann, M. (2011). Andromeda: A Peptide Search Engine Integrated into the MaxQuant Environment. J. Proteome Res. *10*, 1794–1805.

Harding, J.W., and Sneddon, I.N. (1945). The elastic stresses produced by the indentation of the plane surface of a semi-infinite elastic solid by a rigid punch. Math. Proc. Camb. Philos. Soc. *41*, 16–26.

Iuliano, A., van der Wal, E., Ruijmbeek, C.W.B., in ‘t Groen, S.L.M., Pijnappel, W.W.M.P., de Greef, J.C., and Saggiomo, V. (2020). Coupling 3D Printing and Novel Replica Molding for In House Fabrication of Skeletal Muscle Tissue Engineering Devices. Adv. Mater. Technol. *5*, 2000344.

Madden, L., Juhas, M., Kraus, W.E., Truskey, G.A., and Bursac, N. (2015). Bioengineered human myobundles mimic clinical responses of skeletal muscle to drugs. ELife.

Tyanova, S., Temu, T., Sinitcyn, P., Carlson, A., Hein, M.Y., Geiger, T., Mann, M., and Cox, J. (2016). The Perseus computational platform for comprehensive analysis of (prote)omics data. Nat. Methods *13*, 731–740.

van der Wal, E., Bergsma, A.J., van Gestel, T.J.M., in ‘t Groen, S.L.M., Zaehres, H., Araúzo-Bravo, M.J., Schöler, H.R., van der Ploeg, A.T., and Pijnappel, W.W.M.P. (2017). GAA Deficiency in Pompe Disease Is Alleviated by Exon Inclusion in iPSC-Derived Skeletal Muscle Cells. Mol. Ther. - Nucleic Acids *7*, 101–115.
