# Supplementary material for: Highly contractile 3D tissue engineered skeletal muscles from human iPSCs reveal similarities with primary myoblast-derived tissues
Source: Stem Cell Reports. 2023 Sep 28;18(10):1954–71. doi: 10.1016/j.stemcr.2023.08.014 (PMC10656354; doi:10.1016/j.stemcr.2023.08.014)
Supplement: Document S2. Article plus supplemental information [file mmc2.pdf]

Stem Cell Reports
Resource
OPEN ACCESS

Highly contractile 3D tissue engineered skeletal muscles from human iPSCs reveal similarities with primary myoblast-derived tissues

Erik van der Wal,[1,9] Alessandro Iuliano,[2,3,4,9] Stijn L.M. in 't Groen,[2,3,4] Anjali P. Bholasing,[2,3,4] Dominik Priesmann,[5,6] Preeti Sharma,[7] Bianca den Hamer,[1] Vittorio Saggiomo,[8] Marcus Krüger,[5,6] W.W.M. Pim Pijnappel,[2,3,4,10,*] and Jessica C. de Greef[1,10,*]

[1]Department of Human Genetics, Leiden University Medical Center, 2333 ZA Leiden, the Netherlands
[2]Department of Clinical Genetics, Erasmus University Medical Center, 3015 GE Rotterdam, the Netherlands
[3]Department of Pediatrics, Erasmus University Medical Center, 3015 GE Rotterdam, the Netherlands
[4]Center for Lysosomal and Metabolic Diseases, Erasmus University Medical Center, 3015 GE Rotterdam, the Netherlands
[5]Institute of Genetics and Cologne Excellence Cluster on Cellular Stress Responses in Aging-Associated Diseases (CECAD), University of Cologne, Cologne, Germany
[6]Center for Molecular Medicine, University of Cologne, Cologne, Germany
[7]Physical Chemistry and Soft Matter, Wageningen University and Research, 6708 WE Wageningen, the Netherlands
[8]Department of BioNanoTechnology, Wageningen University and Research, 6708 WG Wageningen, the Netherlands
[9]These authors contributed equally
[10]These authors contributed equally
*Correspondence: w.pijnappel@erasmusmc.nl (W.W.M.P.P.), j.c.de_greef@lumc.nl (J.C.d.G.)
https://doi.org/10.1016/j.stemcr.2023.08.014

SUMMARY

Skeletal muscle research is transitioning toward 3D tissue engineered *in vitro* models reproducing muscle's native architecture and supporting measurement of functionality. Human induced pluripotent stem cells (hiPSCs) offer high yields of cells for differentiation. It has been difficult to differentiate high-quality, pure 3D muscle tissues from hiPSCs that show contractile properties comparable to primary myoblast-derived tissues. Here, we present a transgene-free method for the generation of purified, expandable myogenic progenitors (MPs) from hiPSCs grown under feeder-free conditions. We defined a protocol with optimal hydrogel and medium conditions that allowed production of highly contractile 3D tissue engineered skeletal muscles with forces similar to primary myoblast-derived tissues. Gene expression and proteomic analysis between hiPSC-derived and primary myoblast-derived 3D tissues revealed a similar expression profile of proteins involved in myogenic differentiation and sarcomere function. The protocol should be generally applicable for the study of personalized human skeletal muscle tissue in health and disease.

INTRODUCTION

Skeletal muscle tissue generates contractile force providing support for posture and locomotion and enabling respiration. Neuromuscular disorders compromise contractile function of skeletal muscle with muscle weakness and wasting as a result (Benarroch et al., 2019). Drug screening for development of novel therapies is traditionally performed in 2D monolayer cultures or animal models. These models are limited in functional readouts, only partially recapitulate disease phenotypes, and show species-specific drug responses contributing to the high failure rate (~88%) of potential therapeutic strategies in phase I clinical trials (DiMasi et al., 2016).

To improve disease modeling and develop novel therapies, 3D tissue engineered skeletal muscles (3D-TESMs) have been generated from human primary myoblasts allowing functional readout of contractile force, and upon treatment with drugs these 3D-TESMs show similarity with clinical observations (Madden et al., 2015). 3D-TESMs for myasthenia gravis, Pompe disease, and Duchenne muscular dystrophy were recently generated (Afshar Bakooshli et al., 2019; Ebrahimi et al., 2021; Wang et al., 2021), showing the potential for improved disease modeling, drug screening, and the generation of more complex models. However, primary cultures are known for their variability and limited self-renewal, lose myogenic potential after prolonged culturing, and obtaining myogenic cells from aged patients can be challenging (Bigot et al., 2008; Day et al., 2010).

Human induced pluripotent stem cells (hiPSCs) provide unlimited amounts of cells for differentiation (Takahashi and Yamanaka, 2016). So far, only a few studies generated 3D-TESMs using hiPSC-derived muscle cells (Chal et al., 2016; Iuliano et al., 2020; Jiwlawat et al., 2017; Maffioletti et al., 2018; Rao et al., 2018; Selvaraj et al., 2019; Xu et al., 2019). Tissues of hiPSC-derived mesangioblasts that were induced with *MyoD* transgene overexpression formed multinucleated myotubes in 3D, but were unresponsive to electrical stimulation (Maffioletti et al., 2018). Ectopic expression of the muscle stem cell marker *PAX7* in hiPSCs produced myogenic cells with high expansion capacity (Darabi et al., 2012), and 3D-TESMs generated showed responsiveness to electrical stimulation. However, specific tetanic forces generated by these hiPSC-derived 3D-TESMs were lower (3 mN/mm^2) than those of human primary

 © 2023 The Authors.
This is an open access article under the CC BY license (http://creativecommons.org/licenses/by/4.0/).

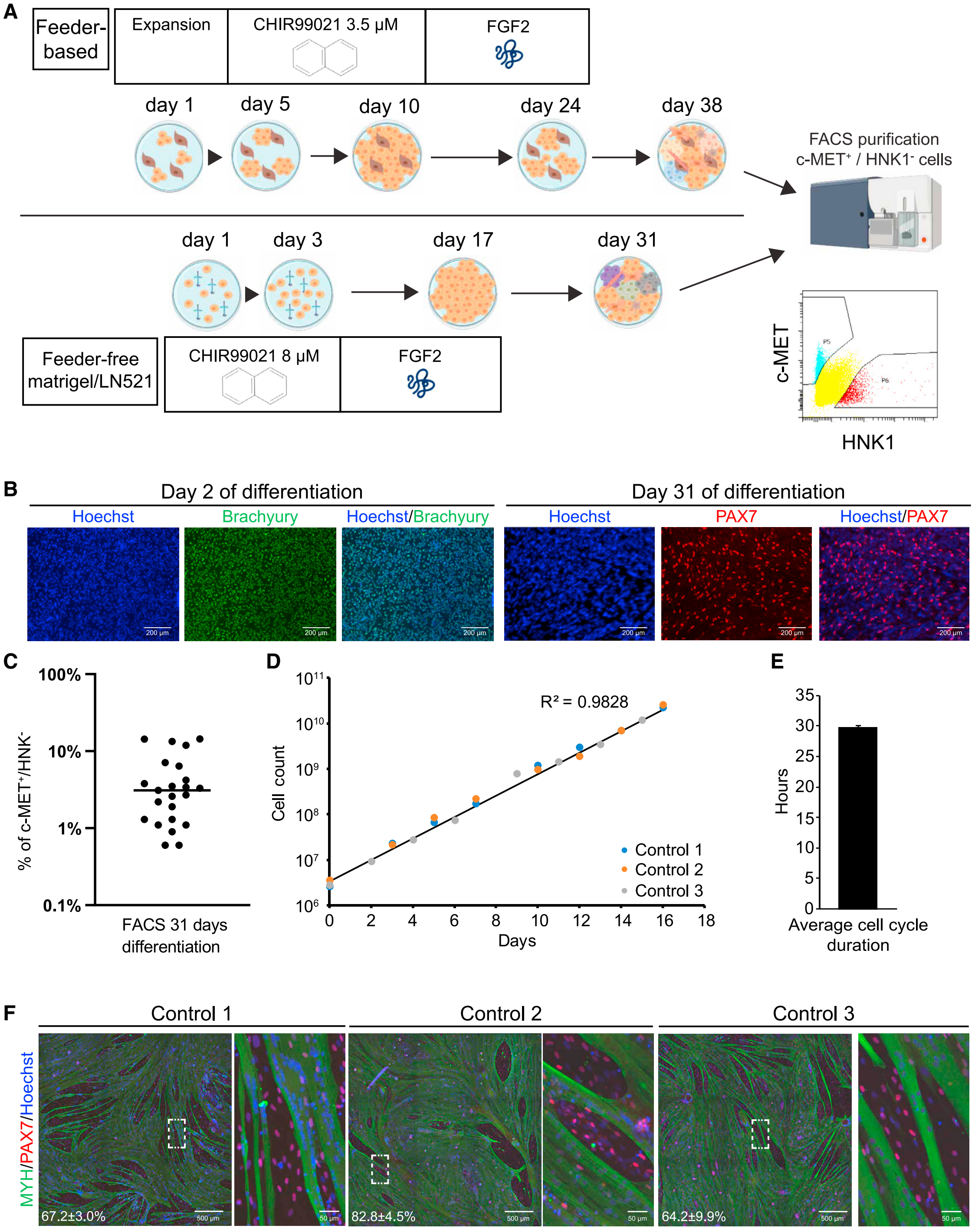

Figure 1. Generation of myogenic progenitors under feeder-free conditions

(A) Overview of the differentiation procedure. Top: feeder-based protocol as published previously (van der Wal et al., 2017). Bottom: feeder-free approach.

(B) Expression of the early mesoderm marker Brachyury (green) and muscle stem cell marker PAX7 (red) analyzed by staining after 2 or 31 days of differentiation using the feeder-free differentiation protocol. Nuclei are stained with Hoechst.

(C) Percentages of c-MET$^+$/HNK$^-$ fraction after 31 days of differentiation showing average ± standard deviation (SD) of 23 independent differentiations.

(legend continued on next page)

myoblast-derived 3D-TESMs (12 mN/mm^2) (Rao et al., 2018). Similarly, myofiber diameter was 9 μm for hiPSC-derived 3D-TESMs and 15 μm for human primary myoblast-derived 3D-TESMs (Madden et al., 2015; Rao et al., 2018). Using endothelial growth medium for differentiation of 3D-TESMs derived from *PAX7*-induced cells increased specific tetanic forces to 33 mN/mm^2, but myofiber diameter was not increased (9 μm) (Xu et al., 2019).

Forcing hiPSCs into myogenic cells via transgene expression might disturb normal physiological processes and reduce maturation capacity. So far, the expansion capacity of myogenic cells generated with the current transgene-free protocols is limited and a few studies generated transgene-free hiPSC-derived 3D-TESMs. However, these studies used unpurified myogenic cells and only Osaki et al. reported functionality with low specific tetanic forces of 0.25 mN/mm^2 (Chal et al., 2016; Jiwlawat et al., 2017; Maffioletti et al., 2018; Osaki et al., 2018). Moreover, there is a consensus on the inferior developmental stage of hiPSC-derived cells compared with primary ones, limiting utilization in disease modeling. We recently showed that purified transgene-free myogenic progenitors (MPs) derived from feeder-based hiPSCs can be expanded up to 5 × 10^{11}-fold, obtaining 10 billion cells from a single well of a six-well plate in less than 10 days of culture, while retaining differentiation capacity in 2D (van der Wal et al., 2017; 2018). We also developed a pipeline for the fabrication of skeletal muscle tissue engineering devices using a simple 3D printing platform generating hundreds of devices a day without use of specialized equipment (Iuliano et al., 2020).

In this study, we adapted our previous myogenic differentiation protocol to feeder-free conditions. We optimized medium and hydrogel composition resulting in 3D-TESMs with high density of titin/dystrophin$^+$ myofibers that are functional. Miniaturization of 3D-TESMs increased density and myofiber size, allowing higher throughput. Treatment of 3D-TESMs with drugs that affect contractile functions showed responses similar to those reported in animal models or humans. When we directly compared 3D-TESMs from multiple hiPSC donors with 3D-TESMs generated from human primary myoblasts, we found that myofiber diameter was similar and specific forces of hiPSC-derived 3D-TESMs were higher than of primary myoblast-derived 3D-TESMs. Proteomic analysis showed a large overlap of proteins that were expressed upon differentiation between donors. The protocol we developed supports consistent and robust formation of high-quality 3D-TESMs from transgene-free hiPSC-derived MPs.

RESULTS

MP generation with feeder-free hiPSC culture

We previously reported the generation of expandable myogenic progenitor lines from feeder-based hiPSCs with a protocol based on Borchin et al. (2013) that consists of three steps (Figure 1A top). The protocol starts with GSK3β inhibition to induce Wnt signaling, followed by FGF2 stimulation and maturation in minimal medium. Thereafter, MPs are purified by fluorescence-activated cell sorting (FACS) for c-MET$^+$/HNK$^-$ cells (van der Wal et al., 2017, 2018). As this protocol includes co-culture of hiPSCs with feeder mouse embryonic fibroblasts, we modified the protocol by adapting it to feeder-free culturing of hiPSCs. Feeder-free hiPSC cultures are widely applied and have improved consistency, allow robust single-cell expansion, and are less labor intensive. To start differentiation, we seeded single hiPSCs, which were stimulated the next day with a high dose of CHIR99021 for 2 days, thereby reducing the differentiation duration of the original protocol with 7 days (Figure 1A bottom). On day 2 of differentiation, virtually all cells expressed the early mesoderm marker Brachyury; on day 31 of differentiation, we detected PAX7-positive patches indicating successful differentiation into muscle stem cells (Figure 1B). FACS analysis of 23 independent hiPSC differentiations yielded an average of 4.6% ± 4.5% of cells that were c-MET$^+$/HNK$^-$ (Figure 1C). Differentiation of two hiPSC donors with either the feeder-based or feeder-free protocol resulted in a similar percentage of c-MET$^+$/HNK$^-$ cells after myogenic differentiation (data not shown). We selected three validated control hiPSC lines (Control 1–3) (Buijsen et al., 2018; van der Wal et al., 2019), generated MPs, and tested their expansion and differentiation capacity. Similar proliferation rates with linear exponential growth were observed for all lines (Figure 1D). Average cell cycle duration was comparable to our previous study with 29.8 ± 0.3 h (van der Wal et al., 2018) (Figure 1E). We next induced differentiation of confluent myogenic progenitor cultures and detected formation of myosin heavy chain (MYH)-positive multinucleated cells (Figure 1F). Fusion rate of MPs was highly dependent on cell number (Figure S1A). Morphology of fused skeletal muscle cells was

(D) Average proliferation curve of myogenic progenitors from controls 1–3 during expansion culture. R^2 was calculated from all data points.
(E) Average cell cycle ±SD of control 1–3 myogenic progenitors during expansion culture derived from (D).
(F) Staining of MYH, PAX7 or Hoechst after 4 days of differentiation of control 1–3 myogenic progenitors. Fusion index is shown in bottom corner as average percentage of nuclei inside MYH-positive cells ±SD and was quantified from five random fields.

homogeneous between the control lines with high fusion indexes (>80%). Notably, fusion index was maintained relatively constant even at higher passages, with control lines 1 and 2 showing a minimal decrease and control line 3 having its fusion index virtually unchanged from passage nine to passage 15 (Figure S1B). Moreover, percentage of $Pax7^+$ cells in culture of differentiated myotubes remained relatively stable at later passages (>4%), with only line 2 seeing a relevant decline at p15 (Figure S1C). These results show that we successfully adapted the myogenic differentiation protocol to feeder-free culture conditions and this protocol robustly generated MPs from different donors with maintained differentiation capacity at late passages.

3D-TESMs from transgene-free hiPSC-derived MPs

Several transgene-free myogenic differentiation protocols exist, but to date no study has shown formation of functional 3D-TESMs from purified transgene-free hiPSCs. To determine whether purified MPs can form 3D-TESMs and to establish the optimal differentiation condition, we investigated four medium compositions reported to support skeletal muscle differentiation (Afshar Bakooshli et al., 2019; Borchin et al., 2013; Rao et al., 2018; van der Wal et al., 2018) (Figure 2A). Previously, we developed a pipeline for fabrication of skeletal muscle tissue engineering devices using a simple 3D printing platform that can generate hundreds of PDMS devices equipped with flexible pillars (Afshar et al., 2020; Hansen et al., 2010; Sakar et al., 2012; Vandenburgh et al., 2008) per day without use of specialized equipment (Iuliano et al., 2020). First, we focused on the Direct Peeling platform, which is the simplest to fabricate and consists of a standard replica molding of PDMS chips from a 3D printed negative mold. We seeded 6×10^5 MPs in a 50-μL hydrogel mixture consisting of 4 mg/mL fibrinogen and 20% Matrigel and casted the hydrogel between two flexible PDMS pillars (Figure 2A). After 2 days, we induced differentiation for 7 days. We detected millimeter-long myofibers at day 7 with a striated pattern of the maturation marker titin in all conditions (Figure 2B). Conditions 1 and 2 revealed a mixture of larger and smaller fibers combined with round contracted cells. In condition 3, we detected abnormal fusion with myofiber branching. Condition 4 displayed elongated myofibers with a homogeneous size and structure. We performed immunostaining on cryopreserved cross-sections to determine the distribution of myofibers inside the 3D-TESMs. Cross-sectional area (CSA) was not significantly different among the four conditions (Figure S2A), and we observed similar-sized myofibers that were double-positive for titin and dystrophin expression in all conditions (Figures S2B and S2C). From this initial morphological evaluation, we overall considered condition 4 to be the ideal one in the generation of 3D-TESMs.

Contractile functionality of 3D-TESMs

To test if 3D-TESMs in these four conditions could show differences on a functional level, we proceeded to test their contractile capacity. MYH composition and sarcomere formation impact contractile force production of skeletal muscle cells (Racca et al., 2013). As conditions 1–4 resulted in efficient myofiber formation upon differentiation, 3D-TESMs were electrically stimulated, and pillar displacement was tracked with high-speed video imaging (Figure 3A). Contractile force (in mN) was then calculated using the average stiffness of PDMS of the Direct Peeling platform (1.59 ± 0.27 MPa) (Figures S3A and S3C), displacement of the pillar, and actual position of the 3D-TESM on the pillar (Figures 3B and 3C) (Legant et al., 2009). 3D-TESMs in all conditions were functional and showed a single twitch contraction when stimulated with a frequency of 1 Hz and a maximum tetanic contraction reaching plateau at a frequency of 20 Hz (Figures 3D and 3E). Doubling the frequency to 40 Hz did not result in increased strength of the tetanic contraction (Figure S3D). Conditions 1–2 showed a similar contractile force, addition of horse serum in condition 3 resulted in a 2-fold increase in absolute and specific forces (forces normalized for CSA) as compared with conditions 1–2 (Figures 3F and 3G). Finally, condition 4 containing KOSR gave the highest specific forces (5.2 ± 2 mN/mm^2 for twitch; 11.1 ± 2.6 mN/mm^2 for tetanus), in agreement with our previous findings (van der Wal et al., 2018). Collectively, 3D-TESMs generated with conditions 1–4 developed into functional, contractile muscle tissues, with condition 4 resulting in 3D-TESMs with the highest quality and contractile forces and therefore being chosen for further experiments.

Defining hydrogel composition for 3D-TESM compaction

After defining the optimal differentiation condition, we examined whether varying the hydrogel concentration could improve density and distribution of myofibers inside 3D-TESMs. A hydrogel containing 4 mg/mL fibrinogen (Afshar et al., 2020; Afshar Bakooshli et al., 2019; Madden et al., 2015; Rao et al., 2018) or more (Selvaraj et al., 2019; Xu et al., 2019) with 20% v/v Matrigel is commonly used for 3D-TESMs. To determine the optimal fibrinogen concentration, we generated 3D-TESMs using final concentrations between 0.5 and 6 mg/mL. Morphology of skeletal muscle cells and myofiber diameter were not significantly changed (Figures S4A and S4B). Myofiber density, however, increased at lower fibrinogen concentrations showing the highest density for the 1-mg concentration with 1,553 ± 191 myofibers positive for dystrophin per mm^2 (Figure S4C). CSA was significantly reduced in 3D-TESMs containing 1 and 0.5 mg/mL of fibrinogen compared with higher concentrations (Figures 4A and 4B). Both absolute

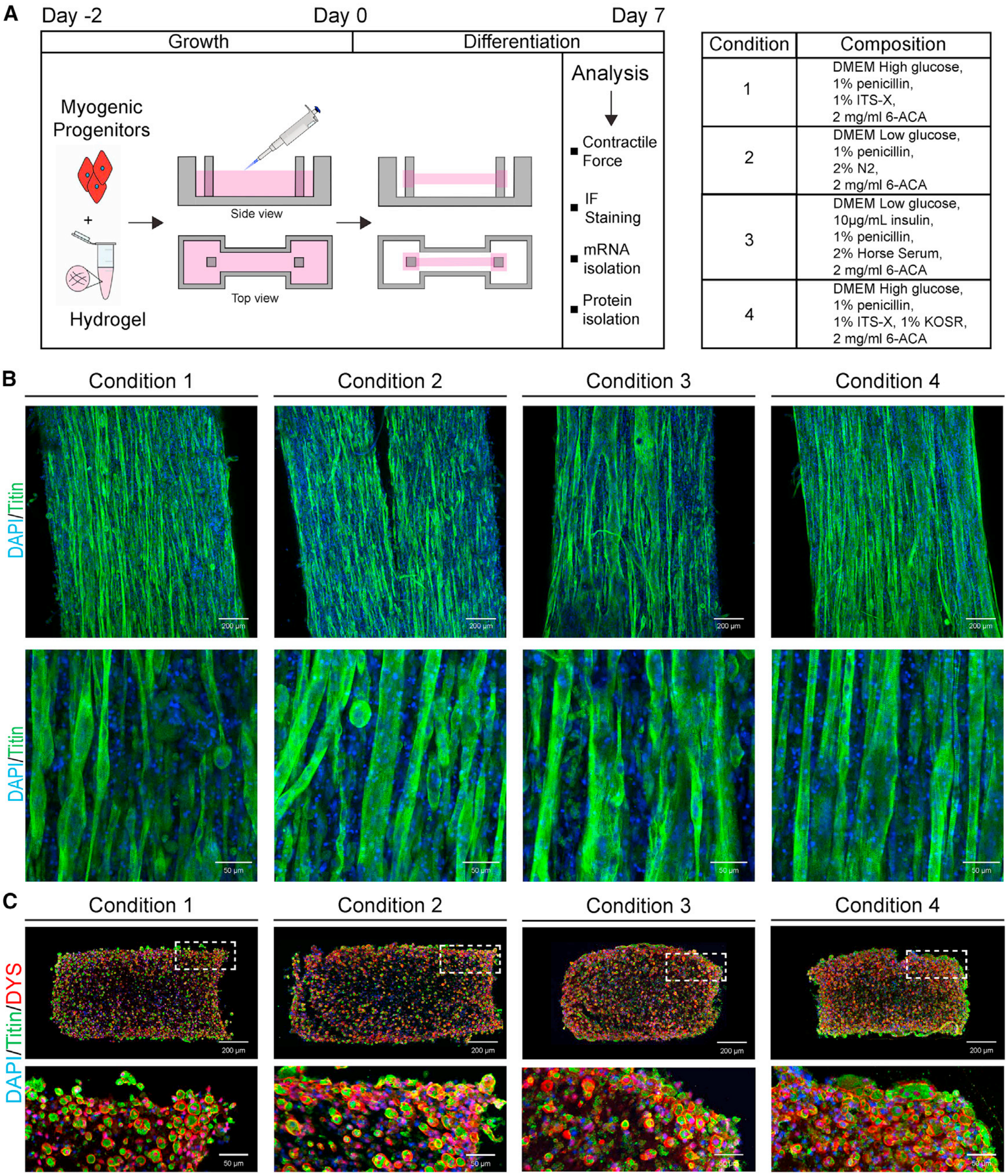

Figure 2. Engineering of 3D-TESMs

(A) Left: Schematic overview of experimental procedure and cartoon illustrating hydrogel compaction in Direct Peeling platform. Right: Overview of four culture conditions used.

(B) 3D-TESMs in the Direct Peeling platform were cultured using conditions 1–4 and stained for titin (green) with whole-mount staining on day 7 of differentiation. Nuclei were visualized with DAPI (blue).

(C) Immunofluorescent staining of cross-sections of 3D-TESMs visualized in (B) with antibodies against titin (green) and dystrophin (red), and counterstained with DAPI (blue).

and specific contractile forces were concentration-dependent and increased with lower fibrinogen concentrations. The highest absolute and specific tetanic forces were detected at a concentration of 1 mg/mL (Figures 4C and 4D). Interestingly, a fibrinogen concentration of 0.5 mg/mL resulted in the highest twitch force, but tetanic

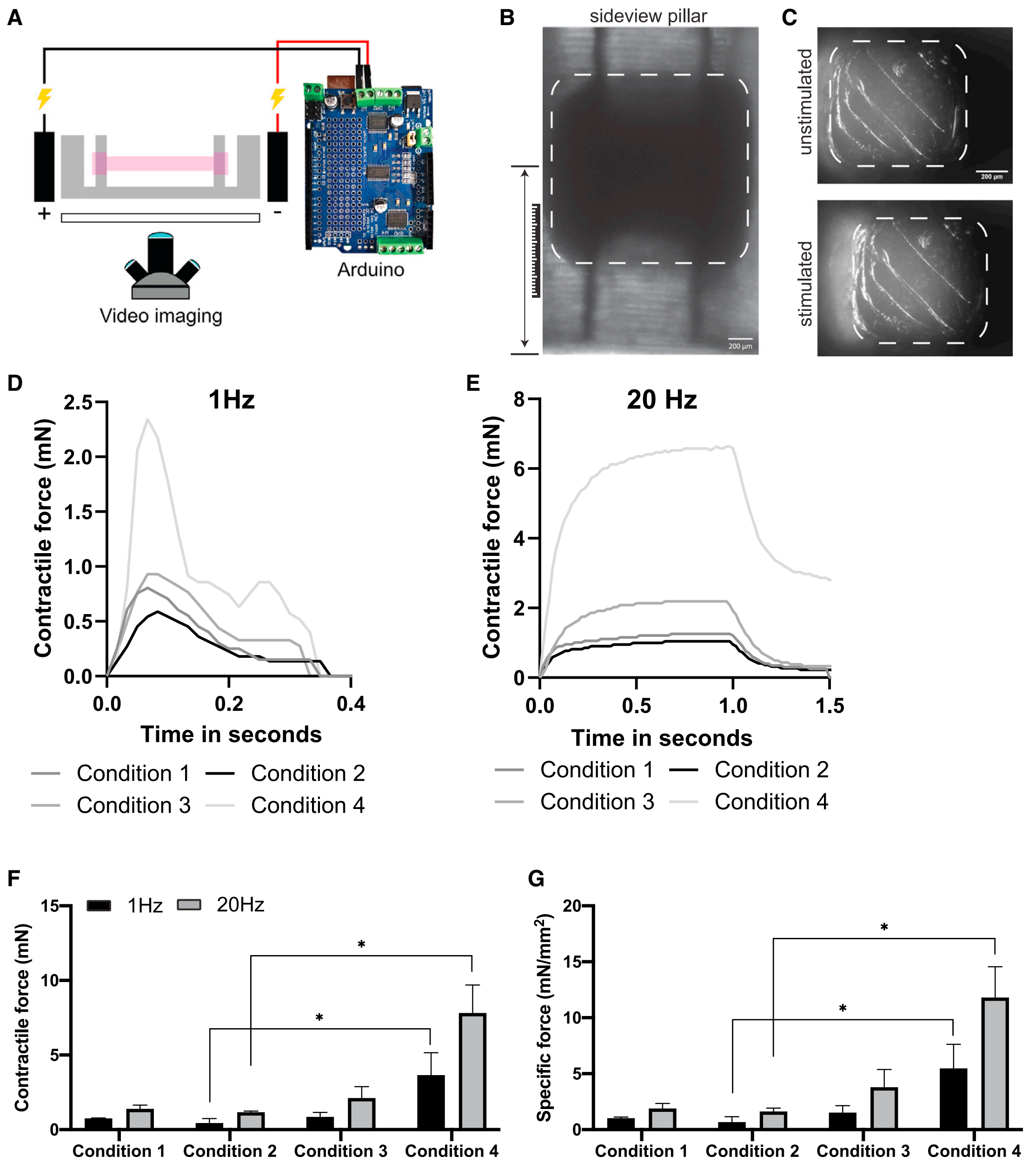

Figure 3. Functional analysis of 3D-TESMs

(A) Cartoon showing video-based contractile force measurement using an Arduino coupled with electrodes for stimulation and high-speed video imaging for recording of pillar displacement.

(B) Sideview of a representative pillar of the Direct Peeling platform with a 3D-TESM attached. Position of the 3D-TESM is used for force calculation.

(C) Top view of the pillar before and during 20-Hz stimulation.

(D) Graph plotting displacement of the pillar upon 1-Hz stimulation for conditions 1–4 on day 7 of differentiation.

(E) As (D) but then for a 20-Hz stimulation.

(F) Same as (D) and (E) but then average absolute contractile force ±SD from three independent 3D-TESMs. Black bars indicate 1-Hz stimulation and gray bars 20-Hz stimulation.

(G) Specific force of same 3D-TESMs as in (F) but then corrected for the cross-sectional area. $**p < 0.01$ using one-way ANOVA with Tukey multiple testing correction.

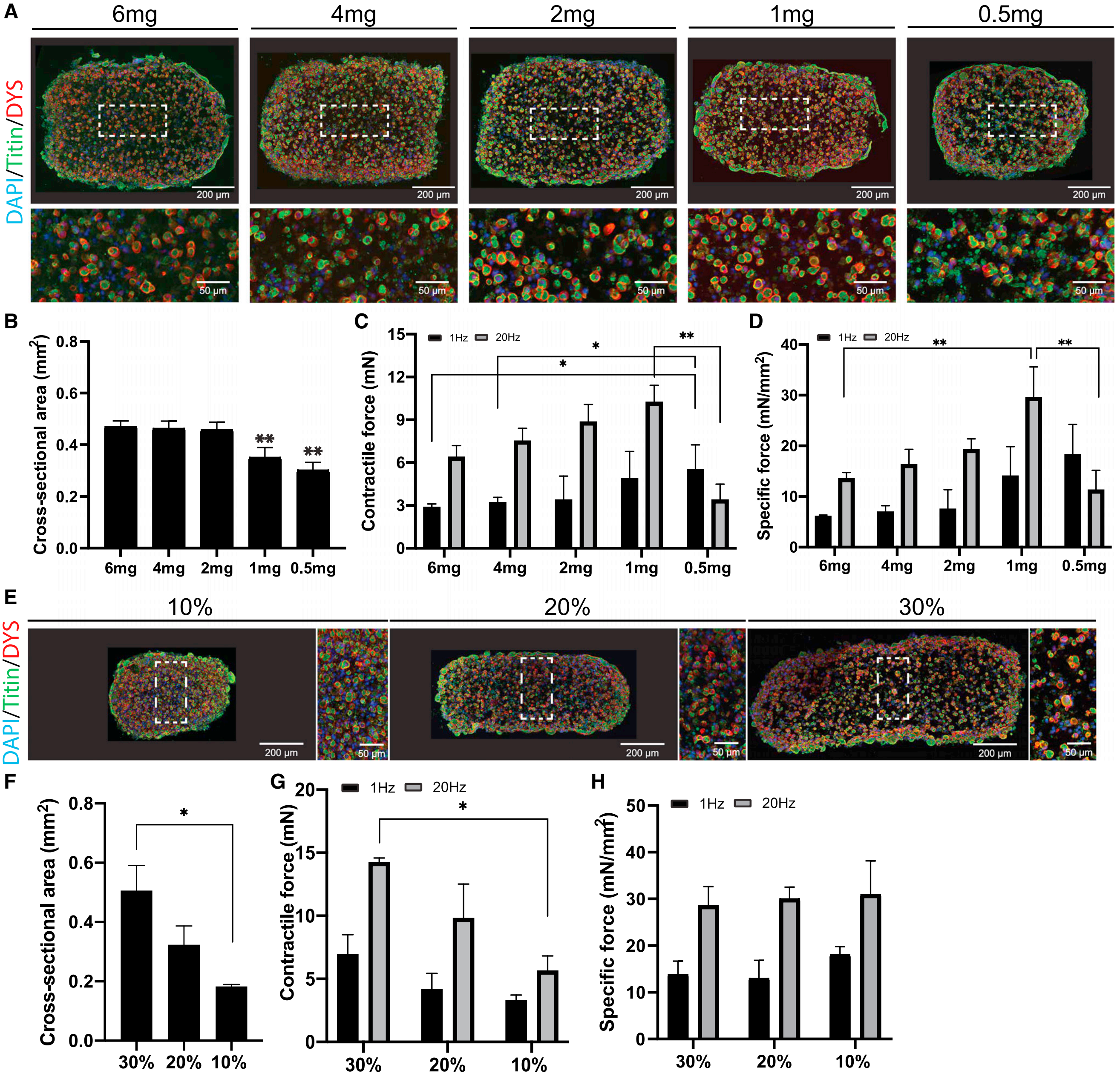

Figure 4. Determination of optimal hydrogel concentration for 3D-TESM formation

(A) Representative cross-sections of 3D-TESMs generated with 6 mg–0.5 mg/mL of fibrinogen in the Direct Peeling platform and analyzed on day 7 of differentiation. Sections were stained with titin (green) and dystrophin (red). Nuclei were visualized in blue by DAPI staining.
(B) Average cross-sectional area of 3D-TESMs generated with different concentrations of fibrinogen.
(C) Average absolute contractile force of 3D-TESMs after stimulation with 1 Hz (black bars) or 20 Hz (gray bars).
(D) Specific force of 3D-TESMs as in (C) but then normalized for cross-sectional area from (B).
(E) Same as (A) but then for 3D-TESMs containing 30%–10% of Matrigel; 1 mg/mL fibrinogen was used.
(F) Average cross-sectional area of 3D-TESMs containing 30%–10% of Matrigel.
(G) Same as (C) but then for Matrigel concentration in 3D-TESMs.
(H) Same as (G) but then corrected for cross-sectional area. $^{*}p < 0.05$, $^{**}p < 0.01$ using one-way ANOVA with Tukey multiple testing correction. Data are derived from three independent 3D-TESMs and expressed as mean ± SD.

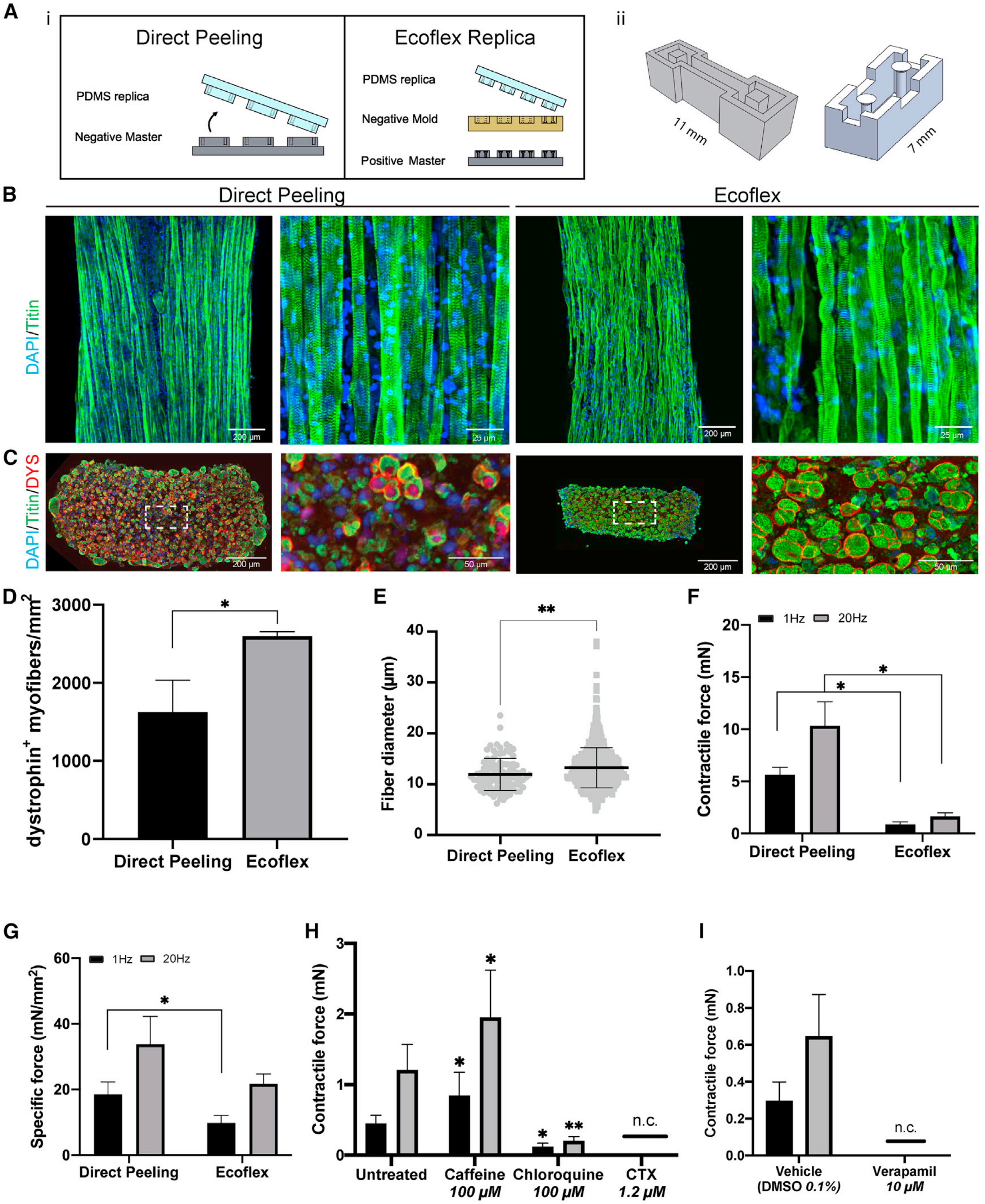

Figure 5. Comparison of the Direct Peeling platform with the Ecoflex Replica platform for miniaturization of 3D-TESMs

(A) Cartoons showing (i) Direct Peeling (left) and Ecoflex Replica platform (right) fabrication schemes, (ii) 3D rendering of Direct Peeling and Ecoflex Replica culture chambers.

(B) Whole-mount staining for titin (green) and nuclei with DAPI (blue) of 3D-TESMs on differentiation day 7.

(C) Cross-sectional staining of a representative 3D-TESM. Antibodies against titin (green) and dystrophin (red) were used combined with DAPI nuclear staining (blue) to visualize myofibers.

(D) Average number of dystrophin^{+} myofibers in cross-sections normalized for cross-sectional area.

(E) Myofiber diameter of n > 100 myofibers (per section) positive for titin/dystrophin measured from n = 3 biological replicas.

(F) Average absolute contractile force of 3D-TESMs stimulated with 1 Hz (black bars) and 20 Hz (gray bars).

(legend continued on next page)

force was significantly decreased as compared with all other concentrations. This result suggests that a minimal fibrinogen concentration is needed to support tetanic contractions in 3D-TESMs. As 1 mg/mL fibrinogen resulted in the highest tetanic force, we used this concentration to subsequently study the impact of different Matrigel concentrations (10%–30%) on 3D-TESM formation. 3D-TESMs generated with fibrinogen alone showed a contractile force that was 10-fold lower than 3D-TESMs with Matrigel included (data not shown). Lowering the Matrigel concentration decreased CSA (Figures 4E and 4F), did not affect myofiber diameter (Figures S4D and S4E), and significantly increased fiber density (Figure S4F). Absolute force reduced when the Matrigel concentration was lowered (Figure 4G). However, upon correction for CSA, specific forces were comparable between all Matrigel concentrations with an average specific force of 14.1 ± 2.6 mN/mm^2 and 28.2 ± 1.1 mN/mm^2 for twitch and tetanic contractions, respectively (Figure 4H). Taken together, lower fibrinogen and Matrigel concentrations in the hydrogel mixture increased fiber density and contractile force of 3D-TESMs. The optimal composition of 1 mg/mL fibrinogen with 20% Matrigel resulted in an ~2-fold increased specific force for both twitch and tetanic contractions when compared with the original hydrogel composition.

Comparison of Direct Peeling and Ecoflex Replica platforms

Drug testing is one of the most promising applications of *in vitro* engineered tissues. We therefore determined if the optimizations defined for the Direct Peeling platform were also suitable for smaller-sized 3D-TESMs that facilitate high-throughput studies. We previously developed a system called Ecoflex Replica, a 2-step replica molding using an ultra-soft elastomeric polymer (Ecoflex 30-00) as intermediate negative mold, to produce small chips fitting 48-well plates that support the formation of 3D-TESMs made with 15 μL of hydrogel containing 24 × 10^4 cells (Iuliano et al., 2020) (Figure 5A). To test whether 3D-TESMs formed in the smaller Ecoflex platform could be efficiently employed in further analysis, we compared 3D-TESMs generated with control 1 cells in the Ecoflex platform to Direct Peeling platform 3D-TESMs. Like before, the position of 3D-TESMs on the pillar was quantified and displacement was recorded upon electrical stimulation (Figure S3E). PDMS stiffness in the Ecoflex platform was comparable with that measured for the Direct Peeling platform (Figures S3B and S3C). Although all conditions were similar between the platforms, we observed that a 1 mg/mL fibrinogen concentration resulted in visible loss of structural integrity in the Ecoflex platform (data not shown) and thus we used a concentration of 2 mg/mL fibrinogen in both platforms and showed millimeter-long titin-positive myofibers on day 7 of differentiation (Figures 5B, S4G, and S4H). Cross-sections stained for titin and dystrophin demonstrated high numbers of double-positive myofibers in both platforms, whereas tissues formed in the Ecoflex platform revealed an increased density of myofibers in the center of 3D-TESMs (Figure 5C). Concordantly, we detected a 1.5-fold increase in the number of dystrophin$^+$ myofibers per mm^2 in the Ecoflex platform relative to the Direct Peeling platform (Figure 5D). Myofiber diameter was only slightly but significantly increased in 3D-TESMs generated in the Ecoflex platform: with a diameter of 13.2 ± 3.9 versus 11.9 ± 3.2 of the Direct Peeling platform (Figure 5E). Next, we stimulated 3D-TESMs and measured a higher absolute contractile force for 3D-TESMs cultured in the Direct Peeling platform (Figure 5F). However, after normalization for CSA specific forces were similar between both platforms with an average of 35.8 ± 0.9 mN/mm^2 for the Direct Peeling platform and 24.5 ± 4.4 mN/mm^2 for the Ecoflex platform (Figure 5G). In conclusion, smaller 3D-TESMs showed a higher myofiber density with a larger diameter while the specific contractile force was only minimally lower compared with bigger tissues. As the Ecoflex platform requires less hydrogel and fewer cells per number of samples, we chose this platform for our final experiments.

Drug testing of 3D-TESMs

We next studied the response of 3D-TESMs to administration of small molecules that are known to affect skeletal muscle functionality. To test whether we could modulate an increase in contractile force, we incubated 3D-TESMs for 6 h with caffeine. Caffeine binds to the ryanodine receptor RyR1, located on the sarcoplasmic reticulum, thereby facilitating Ca^{2+} uptake into muscle fibers and slowing down its reuptake, resulting in enhanced contraction capacity (Neyroud et al., 2019). Indeed, we detected an increase in twitch and tetanic contractile forces of 3D-TESMs after incubation with caffeine compared with untreated 3D-TESMs (Figure 5H). Interestingly, the effects of

(G) Same as (F) but then specific force (normalized for cross-sectional area).
(H) Average absolute contractile force of 3D-TESMs on day 7 of differentiation in Ecoflex platform after 6 h of incubation with caffeine, chloroquine, or cardiotoxin (CTX). 3D-TESMs were stimulated with either 1 Hz (black bars) or 20 Hz (gray bars).
(I) Same as (H) but then for 3D-TESMs treated for 1 h with verapamil. N.C. in (H) and (I) stands for not contractile. *$p < 0.05$, **$p < 0.01$, ****$p < 0.0001$ using either independent-samples t test (D–G) or one-way ANOVA with Tukey multiple testing correction (H). Data are derived from three (D–G) or six to 10 (H and I) independent 3D-TESMs and expressed as mean ± SD.

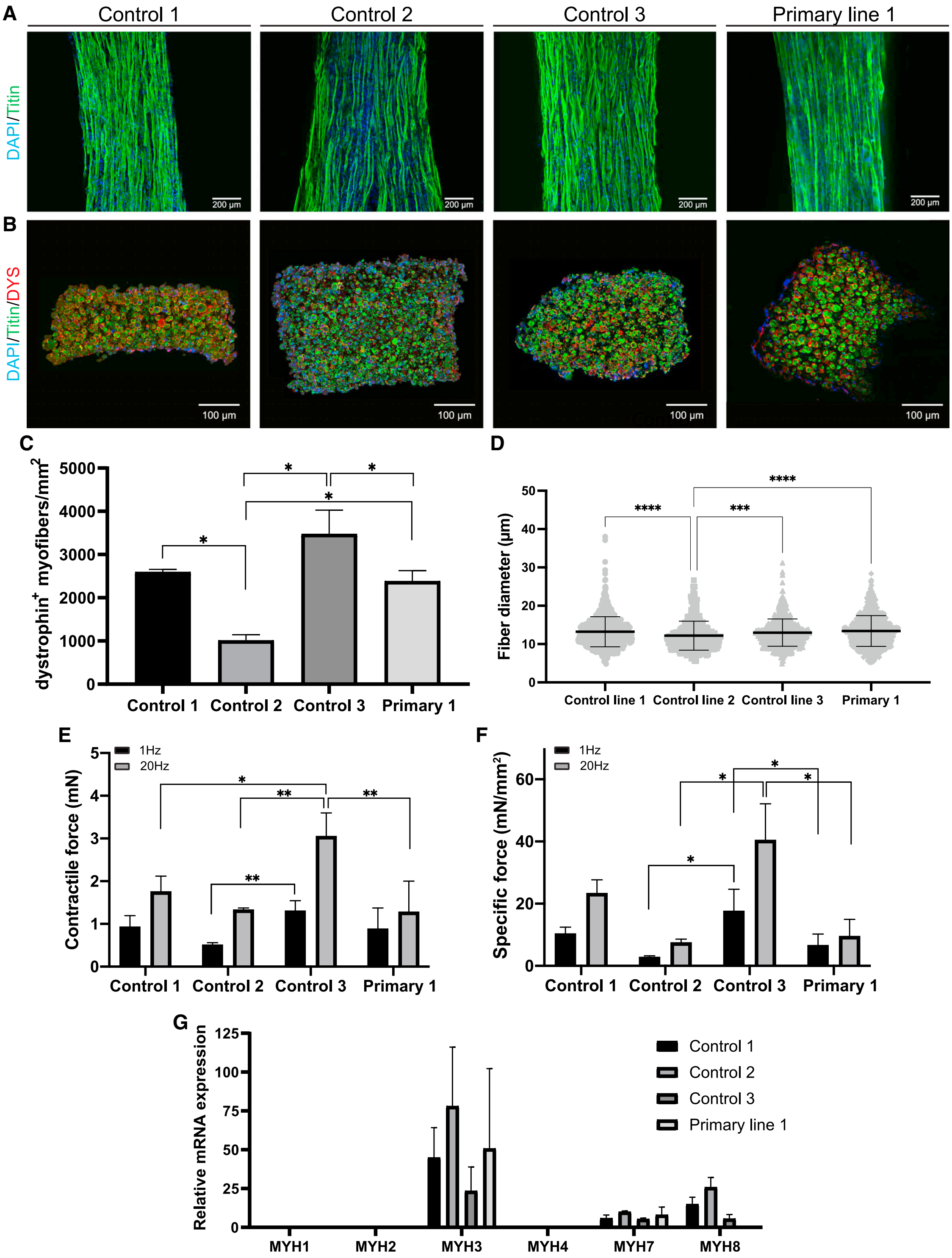

Figure 6. 3D-TESM generation of multiple donors and comparison with 3D-TESMs generated from primary myoblasts

(A) 3D-TESMs of control 1–3 myogenic progenitors and primary myoblasts in the Ecoflex platform were stained for titin (green) and nuclei were visualized with DAPI (blue) on day 7 of differentiation.

(B) Titin (green) and dystrophin (red) labeling of cross-sections generated from control 1–3 3D-TESMs or 3D-TESMs from primary myoblasts line 1. Nuclei were stained with DAPI (blue).

(legend continued on next page)

caffeine on contractile force showed a certain variability across different lines and concentrations, with lines 1 and 3 responding positively already after 1 h and also with lower glucose in the medium, while line 2 showed no apparent response (Figures S5C and S5D). Chloroquine can induce myopathies characterized by myofiber vacuolization, resulting in general muscle weakness in treated patients (Carvalho, 2020). Treatment with chloroquine resulted in a ~3-fold decrease for twitch contractions and a ~5-fold decrease for tetanic contractions (Figures 5H, S5C, and S5D). Cardiotoxin (CTX) is a protein extracted from snake venom with a potent cytotoxic effect on skeletal muscle cells, as it induces lysis of the sarcolemma, with hypercontraction of sarcomeres and myonecrosis as a result (Kaur et al., 2012). Treatment with CTX resulted in 3D-TESMs unresponsive to electrical stimulation (Figure 5H). Immunostaining of treated 3D-TESMs showed a decrease in myofiber quality, with marked presence of extracellular debris for chloroquine, and revealed a complete disruption of internal architecture of 3D-TESMs, with no detectable presence of intact myofibers, for CTX (Figure S5B). Finally, we incubated 3D-TESMs with verapamil, which is an antagonist of RyR1 and tested whether 3D-TESMs can also be used to determine short-term effects on the contractile machinery. After 1 h of treatment, we observed unresponsiveness to electrical stimulation. 3D-TESMs treated with DMSO generated both twitch and tetanic contractions (Figure 5I). In both untreated and verapamil-treated 3D-TESMs, we observed intact fibers within 3D-TESMs and preserved sarcomere structures (Figure S5C). Altogether, these results highlight the sensitivity of 3D-TESMs to drug treatment using relevant concentrations and similar functional responses were observed as in *in vivo* models.

3D-TESM formation of multiple donors and comparison with 3D-TESMs generated from human primary myoblasts

To evaluate the robustness of generating 3D-TESMs from hiPSC-derived MPs, we extended our analysis to control 2–3 MPs. As 3D-TESMs generated from primary myoblasts have so far shown the highest contractile forces (Madden et al., 2015), we also included control primary myoblasts in this comparison. On day 7 of differentiation, we observed striated myofibers positive for titin in 3D-TESMs generated from controls 1, 2, and 3, as well as from primary myoblast lines from three different donors (Figures 6A and S6). Comparison among the three primary lines revealed how primary line 1 showed the highest force, regardless of the medium used for their differentiation (Figures S6A and S6B). Further analyses described here have been made using primary line 1. Cross-sectional analysis revealed similar numbers of dystrophin$^+$ myofibers per mm^2 in control 1 and primary myoblasts, higher numbers in control 3, and 2-fold lower numbers in control 2 (Figures 6B and 6C). Average myofiber diameter was ~13 μm among 3D-TESMs from all four lines with minimal differences across groups (Figure 6D). All myogenic progenitor lines generated functional 3D-TESMs that contracted upon electrical stimulation. Twitch and tetanic contractions for control 1, control 2, and primary myoblasts ranged between 0.5–1 mN and 1–2 mN in absolute force, respectively, while control 3 showed the highest absolute contractile forces with 1 mN for twitch and 3 mN for tetanic contractions (Figure 6E). When normalized for their CSA, lines 1 and 3 reached average specific tetanic forces of 21.6 and 37 mN/mm^2 (SD ±3 and ±7), respectively. 3D-TESMs from control 2 and primary myoblasts showed lower compaction of the hydrogel, and thus specific forces of these 3D-TESMs were 2.5-fold and 4-fold lower than those from control 1 and control 3 3D-TESMs, respectively (Figure 6F). Line 2 reached an average specific tetanic force of 7 mN/mm^2, while tissues from the primary line reached an average of 8.9 mN/mm^2 (SD ±0.99 and ±4.9). We next compared 3D-TESMs generated from MPs and primary myoblasts by profiling mRNA expression of *MYH* isoforms (Figure 6G). In all 3D-TESMs, we detected high expression of embryonic *MYH3* isoform and a lower expression of slow *MYH7* isoform, which is present in both embryonic/fetal and adult skeletal muscle. It is to be mentioned that *MYH7* showed a particular variability in parallel analysis among the other primary lines (Figure S6B). Neonatal *MYH8* isoform was mainly detected in 3D-TESMs derived from MPs and lowly expressed in 3D-TESMs from primary myoblasts, while *MYH1*, *MYH2,* and *MYH4* were undetectable. These *MYH* profiles indicate a fetal/neonatal-like state for both hiPSC-derived and primary myoblast-derived 3D-TESMs. In conclusion, MPs derived from different hiPSC donors formed highly functional 3D-TESMs containing titin and dystrophin$^+$ myofibers with comparable diameter.

(C) Comparison of the number of dystrophin$^+$ myofibers in 3D-TESMs from different lines.
(D) Myofiber diameter of n > 120 myofibers (3–4 cross-sections) quantified from n = 3 tissues per line.
(E) Average absolute contractile force after stimulation with 1 Hz (black bars) and 20 Hz (gray bars).
(F) Same as (E), but then specific force.
(G) Relative mRNA expression of MYH isoforms in 3D-TESMs from control 1–3 myogenic progenitors and primary myoblasts line 1. Data were normalized for GUSB expression. *p < 0.05, **p < 0.01 using one-way ANOVA with Tukey (C) or Games-Howell multiple testing correction (E, F). Data are derived from three (C, D, and G) or three to six (E and F) independent 3D-TESMs and expressed as mean ± SD.

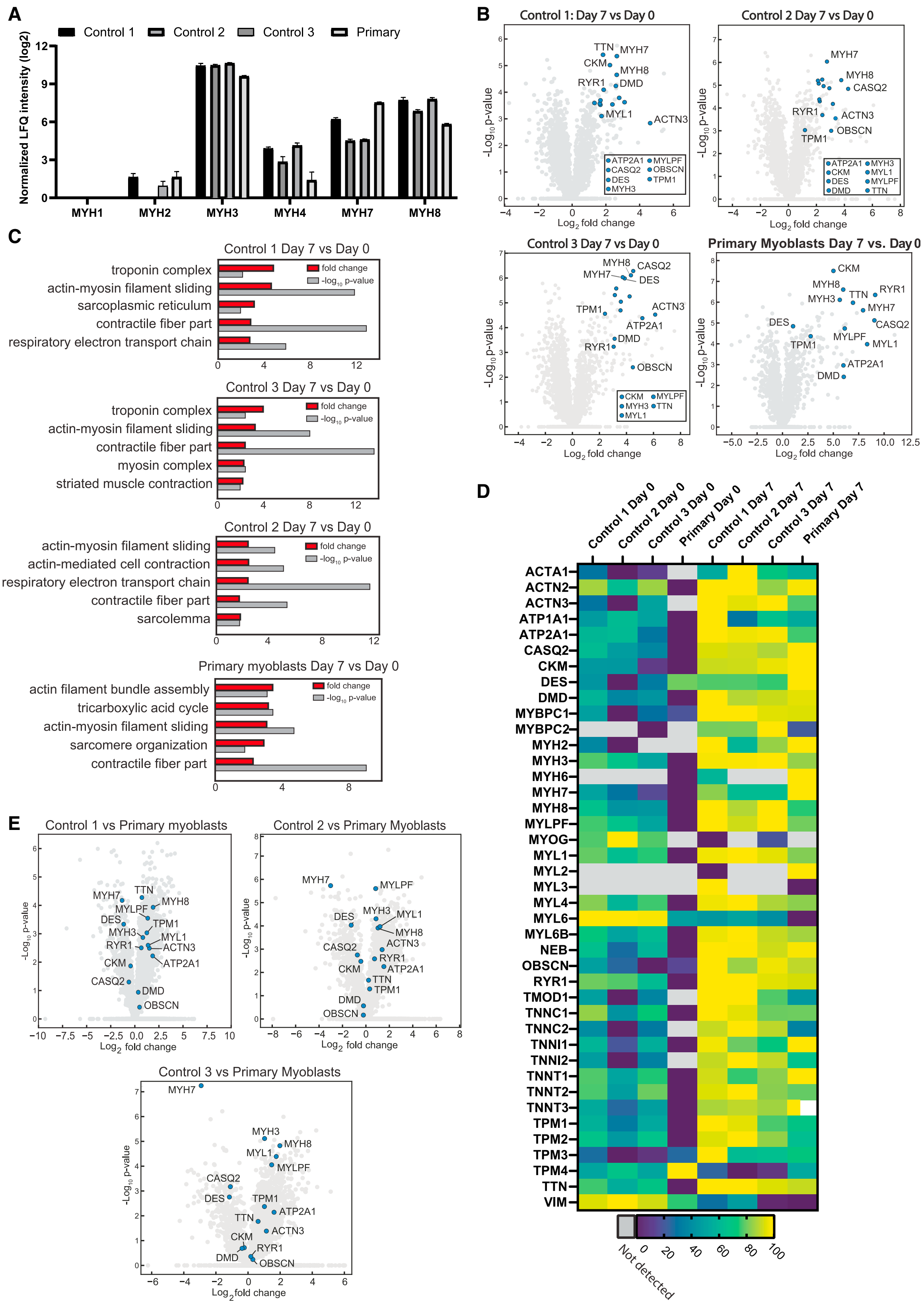

(legend on next page)

3D-TESMs generated from different hiPSC donors showed specific forces of 10–34 mN/mm^2, which is in a similar range as specific forces of 3D-TESMs generated from primary myoblasts (12 mN/mm^2).

Protein expression in hiPSC-derived and primary myoblast-derived 3D-TESMs

We performed proteomic analysis to further characterize and compare 3D-TESMs generated from hiPSC donors and primary myoblasts. We first analyzed MYH isoforms: as found for mRNA expression, MYH3 showed the highest abundance and was expressed at similar levels in all lines (Figure 7A). Interestingly, we observed MYH2 and MYH4 protein expression, which was undetectable at the mRNA level (Figures 6G and 7A), likely due to higher sensitivity of mass spectrometry compared with RT-qPCR. Expression of MYH4 and MYH8 was higher in myogenic progenitor-derived 3D-TESMs compared with primary myoblast-derived, whereas expression of MYH7 was lower. Adult-fast MYH1 protein was not detected at day 0 and day 7 in all four lines. Next, we analyzed the development of myofibers by comparing 3D-TESMs at day 0 with 3D-TESMs at day 7 of differentiation. Of the proteins detected in control 1 3D-TESMs, 692 proteins were significantly upregulated or downregulated, followed by 965 proteins for control 2, 1,303 proteins for control 3, and 801 proteins for primary myoblasts (Figure 7B). Overall, we observed a strong induction of proteins involved in skeletal muscle cell differentiation in all lines on day 7 of differentiation. Proteins commonly associated with skeletal muscle tissue are highlighted in Figure 7B, and this was also evident from plotting protein rank against cumulative intensity-based absolute quantification (iBAQ) intensity (Figure S7). The results were confirmed by gene ontology (GO) enrichment analysis, where most enriched pathways were associated with either development or functioning of skeletal muscle tissue (Figure 7C), with “actin-myosin filament sliding” and “contractile fiber part” being in the top five enriched GOs from all four donors.

Further analysis of most enriched GOs found at least four out of five most enriched GOs to be directly associated with sarcomere in both hiPSC-derived and primary myoblast-derived 3D-TESMs (Figure 7C). Interestingly, primary myoblast-derived 3D-TESMs showed for most proteins a lower expression at day 0, which resulted in the highest fold change when compared with day 0 of differentiation (Figure 7D). While the process of myogenesis was upregulated and comparable between hiPSC-derived and primary myoblast-derived 3D-TESMs, direct comparisons between hiPSC-derived and primary myoblast-derived 3D-TESMs at day 7 of differentiation showed more subtle differences when analyzing proteins associated with different muscle/fiber types, including MYHs, MYLs, and ACTN3 (Figure 7E). Taken together, both hiPSC-derived MPs and primary myoblasts could form highly contractile 3D-TESMs with similar induction of proteins involved in skeletal muscle development and muscle contraction.

DISCUSSION

Here, we adapted our previously described transgene-free myogenic differentiation protocol (van der Wal et al., 2018) to feeder-free conditions, resulting in MPs with similar expansion capacity (Figure 1), high viability after cryopreservation, and efficient differentiation into multinucleated myotubes. In combination with our previous work to produce versatile 3D culture chambers (Iuliano et al., 2020), we showed the formation of high-quality 3D-TESMs from three donors. We optimized culture conditions, characterized morphology, performed functional analysis, tested drug responses, and performed proteomics.

Optimization of 3D culture conditions and hydrogel formulation further improved 3D-TESMs (Figures 2, 3, and 4). Addition of KOSR in differentiation medium showed no significant differences in fiber diameter and density among conditions; however, it provided the highest contractile force (Figures 2 and 3). A hydrogel formulation

Figure 7. Proteomic analysis of hiPSC-derived and primary myoblast-derived 3D-TESMs
(A) Difference in LFQ intensity (normalized by column-wise median subtraction for each sample) between day 0 and day 7 of main MYH isoforms commonly associated with skeletal muscle, detected in control 1–3 hiPSC-derived 3D-TESMs and primary myoblast-derived ones.
(B) Volcano plot for all three control lines and primary 3D-TESMs, showing fold change of all proteins identified at day 7 compared with day 0, based on LFQ intensity. An additional subset of myogenic markers is highlighted in blue and shown in the bottom-right box.
(C) Gene ontology enrichment analysis performed for controls 1–3 and primary 3D-TESMs. Comparisons were made between day 0 and day 7 of differentiation for each line separately. Top five significantly enriched pathways are shown for each respective control.
(D) Heatmap showing normalized LFQ intensity of selected proteins with highest sample set to 100% for each respective protein. Proteins were selected based on their association with development and function of skeletal muscle tissue, for each line at day 0 and day 7 of differentiation.
(E) Volcano plots comparing hiPSC-derived 3D-TESMs with primary myoblast-derived 3D-TESMs. Comparisons of iBAQ intensity of each protein, with a subset of myogenic markers highlighted in blue in the bottom-right box. Data are derived from three independent 3D-TESMs and expressed as mean ± SD or as mean.

with a lower fibrinogen concentration (1–2 mg/mL) also increased contractile force (Figure 4). With a maximum specific tetanic force of ~40 mN/mm^2, the 3D-TESMs produce a specific force that is between the force of fetal (6 mN/mm^2) and adult (84 mN/mm^2) skeletal muscle (Day et al., 2010; Racca et al., 2013), and well above the contractile force described previously (~6 mN/mm^2) for hiPSC-derived 3D-TESMs (Rao et al., 2018; Selvaraj et al., 2019; Xu et al., 2019). Qualitative analysis of cross-sections showed a morphological structure comparable to fetal skeletal muscle of 13–18 gestational weeks, characterized by myofibers dispersed in abundant ECM (Romero et al., 2013). By downsizing 3D-TESMs to increase throughput, the density and diameter of myofibers was slightly increased with an average diameter of ~13 μm (Figure 5), which is larger than for other models (Iuliano et al., 2020; Rao et al., 2018; Xu et al., 2019). Such morphology appears to resemble 20- to 25-week fetal muscle (Romero et al., 2013), but is not close to the ~70-μm myofiber diameter of adult skeletal muscle (Krivickas et al., 2011). This structural improvement in response to downsizing suggests a size threshold for 3D-TESMs, and may be related to general diffusion distance of oxygen and nutrients in tissues (Liu et al., 2015).

Direct comparison of 3D-TESMs generated from human primary myoblasts showed similarities at qualitative, molecular, and functional levels. Interestingly, hiPSC-derived 3D-TESMs produced significantly higher specific contractile forces in two out of three lines (Figures 6E and 6F) compared with primary myoblast-derived ones, which performed in line with reported primary myoblast-derived 3D TESMs (6–12 mN/mm^2) (Afshar et al., 2020; Madden et al., 2015; Mills et al., 2019). Myofiber number, size, and appearance did not differ considerably between 3D-TESMs generated with hiPSC-derived MPs or with primary myoblasts (Figures 6A–6D). Primary myoblast-derived 3D-TESMs followed a similar expression pattern for embryonic *MYH3*, neonatal *MYH8,* and slow type I *MYH7* as hiPSC-derived 3D-TESMs, with some differences in relative expression levels (Figures 7A and 7E). Such small differences are not expected to have a relevant influence on the response of the different cell sources to drugs. It has been shown that *in vitro* myogenic cell cultures switch back to expressing embryonic *MYH3* and type I *MYH7* regardless of the developmental stage of cells (Cho et al., 1993), with some differences between muscle sources (Wehrle et al., 1994). During *in vivo* regeneration of skeletal muscle, newly formed fibers derived from satellite cells express *MYH3*, *MYH7,* and *MYH8* for the first 2–3 weeks, before turning to adult-fast *MYH1* and *MYH2* expression (Esser et al., 1993; Schiaffino et al., 2015; Zhou et al., 2019).

Proteomic analysis of 3D-TESMs of MPs and primary myoblasts showed that all tissues displayed enhanced expression of MYH3, MYH8, and MYH7, relative to undifferentiated cells (Figure 7A). Similar MYH expression patterns were reported by Mills et al., who also performed proteomic analysis on human primary myoblast-derived 3D-TESMs (Mills et al., 2019). The larger increase of expression detected in primary myoblast-derived 3D-TESMs (Figures 7B and 7C) could suggest a more primed state and faster differentiation toward skeletal muscle cells (Figures 7D and 7E). Gene expression and proteomic profiling in this and other studies highlights a yet immature state of 3D-TESMs (Khodabukus, 2021; Mills et al., 2019; Mueller et al., 2021; Rao et al., 2018), compared with *in vivo* tissues. However, from our data we could not find any relevant functional or molecular difference that could justify the preference of primary-derived 3D tissues over hiPSC-derived ones as *in vitro* models. To improve maturation of the tissues, exercise by electrical stimulation and co-culture can be applied (Afshar Bakooshli et al., 2019; Khodabukus, 2021; Khodabukus et al., 2019).

The possibility of obtaining tens of billions of pure MPs from any donor after a single myogenic differentiation (van der Wal et al., 2018), the fast fabrication of PDMS devices (Iuliano et al., 2020), the optimized 3D culture conditions, and resulting high contractile forces found in this study make this *in vitro* model an attractive instrument to study basic biology and disease of skeletal muscle tissue.

The present study aimed at characterizing the functional and molecular properties of differentiated 3D-TESMs obtained from hiPSC-derived MPs. Limitations of this study are as follows. Functional contractile properties have been investigated with a post-deflection approach, which although being today widely used (Vandenburgh, 2008; Hansen, 2010; Sakar, 2012; Afshar, 2020), it is substantially different from the direct force-transducers techniques measuring absolute force and determining optimal force-length relationships. A direct comparison between the two methods is envisioned in future follow-up studies. Moreover, calcium homeostasis is a dynamic mechanism constantly reshaping in developing muscle. Future analysis should take in consideration this important aspect, in relation to its development over an extended culture period and not only to the initial differentiation phase. These considerations are also applicable in the context of drug studies, especially those directly affecting calcium handling by myotubes, as their functional effects may change with respect to the developmental stage. Future studies should extend this characterization to more mature tissues. Finally, we observed considerable differences in contractile properties and responses to drugs between lines derived from different donors. This likely reflects known differences between individuals due to differences in genetic background, although other causes may also apply, including age at biopsy, acquired somatic genetic variants,

and clonal differences between lines. When it comes to disease modeling and testing drugs, the use of isogenic controls is recommended to distinguish disease-related read outs from random variation.

EXPERIMENTAL PROCEDURES

For further details, see supplemental experimental procedures.

Resource availability

Corresponding authors

W.W.M. Pim Pijnappel: w.pijnappel@erasmusmc.nl, Jessica de Greef: J.C.de_Greef@lumc.nl.

Materials availability

Requests for additional raw and analyzed data, as well as materials, will be promptly received and reviewed by the members of the Pijnappel lab and de Greef lab at Erasmus Medical Center and Leiden University Medical Center, respectively, to verify if the request is subject to any intellectual property of confidentiality obligations. Any data and materials that can be shared will be released via a material transfer agreement.

Data and code availability

The mass spectrometry proteomics data have been deposited to the ProteomeXchange Consortium via the PRIDE (Perez-Riverol et al., 2022) partner repository with the dataset identifier (accession number) PXD045145.

Feeder-free culture and differentiation of hiPSCs into MPs

Control 1–3 (LUMC0162iCTRL05, LUMCi003-A, LUMCi023-A) hiPSCs were previously generated (Buijsen et al., 2018; van der Wal et al., 2019). hiPSCs were cultured in mTESR1 medium (STEMCELL technologies) on Matrigel (Corning) or LN521 (BioLamina) coating. For myogenic differentiation, hiPSC cultures were detached with TrypLE express reagent (Gibco) and plated with 5×10^4 cells/mL in mTESR1 medium with 1× RevitaCell supplement (Gibco). The next day, cells were switched to myogenic differentiation medium (DMEM/F12, 1% Penicillin/Streptomycin (p/s), 1% ITS-X, all Gibco) supplemented with 8 μM CHIR99021 (Axon Medchem). On day 3, medium was changed to myogenic differentiation medium with 20 ng/mL FGF2 (Peprotech) and on day 17 to myogenic differentiation medium only. On day 31, cells were detached and labeled with Hoechst (1:10,000, H3569, Thermo Fisher Scientific), α-*c*-MET APC-conjugated (1:50, FAB3582A, R&D Systems) and α-CD57 PE-conjugated (1:100, 12-0577-42, Thermo Fisher Scientific), as described previously (van der Wal et al., 2018). $Hoechst^-$/$c\text{-}MET^+$/$CD57^-$ fraction was collected in FACS-recovery medium (DMEM HG [Gibco], 10% fetal bovine serum [FBS] [Biowest], 1% p/s, 1× RevitaCell supplement and 100 ng/mL FGF2) and plated on ECM-coated dishes (1:200, E6909-5 mL, Sigma-Aldrich). The myogenic progenitor lines employed in this study were obtained from one single differentiation round per hiPSC line.

MP culture and 2D differentiation

MPs were cultured on ECM-coated dishes, expanded in growth medium (GM) consisting of DMEM HG, 1% p/s, 10% FBS, and 100 ng/mL FGF2, and detached using 1:1 diluted TrypLE express reagent with PBS. Differentiation to skeletal muscle cells was induced with differentiation medium (DM) (DMEM HG, 1% Penicillin-G [Sigma-Aldrich], 1% ITS-X and 1% knockout serum replacement [KOSR, Gibco]).

Formation of 3D tissue engineered skeletal muscles of MPs

3D-TESMs generated in Direct Peeling chambers (50 μL hydrogel) contained 60×10^4 cells per tissue and 3D-TESMs in Ecoflex Replica chambers (15 μL hydrogel) 24×10^4 cells per tissue. Hydrogel mixture contained 1 or 2 mg/mL fibrinogen (Sigma-Aldrich), 20% Matrigel growth factor reduced (Corning), and MPs, or as indicated in figures (on ice). Cross-linking of fibrinogen was initiated by adding 0.8 units/mL of Bovine Thrombin (Sigma-Aldrich) and was directly pipetted inside chambers. 3D-TESMs were incubated for 30 min at 37°C before addition of GM supplemented with 1.5 mg/mL 6-aminocaproic acid (6-ACA) (Sigma-Aldrich). After 2 days, differentiation was induced by switching medium to DM supplemented with 2 mg/mL 6-ACA, or as indicated in the figures. Every 48 h half of the medium was refreshed. 3D-TESMs were cultured on a 65 rpm shaking platform at 37°C/5% CO_2.

Force measurements

For electrical stimulations, an Arduino Uno Rev3 equipped with an Adafruit motor shield V2 was used. Carbon plate electrodes were oriented parallel to the major axis of 3D-TESMs. Stimulations were performed at a frequency of 1 or 20 Hz with 2.45 V and a duty cycle of 10%. Displacement of pillars was recorded with a DCC3240M camera (Thorlabs) at 60 frames per second and analyzed with ImageJ for displacement or with a Python script (Afshar et al., 2020). Pillar position of 3D-TESMs was measured via images from the back of the pillar. Forces were calculated with $Force\ in\ N = \frac{Ewt^3}{2a^2(3L-a)}\delta$ for the Direct Peeling platform, and $Force\ in\ N = \frac{6E\pi r^4}{4a^2(3L-a)}\delta$ for the Ecoflex Replica platform using determined stiffness of PDMS for each model (Legant et al., 2009). To calculate specific forces, cross-sections were generated and absolute contractile forces were normalized by CSA.

Statistical analysis

Statistical analysis was performed using GraphPad Prism 8.0 (GraphPad Software, LLC) and SPSS Statistics (IBM). All error bars refer to mean ± SD.

SUPPLEMENTAL INFORMATION

Supplemental information can be found online at https://doi.org/10.1016/j.stemcr.2023.08.014.

ACKNOWLEDGMENTS

The authors would like to thank Dr Philip Lijnzaad (Princess Máxima Center for Pediatric Oncology, Utrecht, Netherlands) and Dr Gerben Schaaf (Department of Clinical Genetics, Department of Pediatrics, Center for Lysosomal and Metabolic Diseases, Erasmus University Medical Center, Rotterdam, Netherlands) for the support regarding statistical analysis.

The collaboration project is co-funded by the PPP Allowance made available by Health-Holland, Top Sector Life Sciences & Health, to the Prinses Beatrix Spierfonds to stimulate public-private partnerships (project numbers LSHM17075, LSHM19015, and LSHM20011). Funding was also obtained from the EU Joint Programming initiative for Neurodegenerative Disease (JPND) Research to the Netherlands Organisation for Health Research and Development (ZonMw) project 733051105.

AUTHOR CONTRIBUTIONS

E.v.d.W. and A.I. contributed equally to this work. W.W.M.P.P. and J.C.d.G. are shared senior authors. E.v.d.W. and A.I. fabricated the devices and designed and performed the experiments. S.L.M.in't.G., P.S., D.P. and B.v.H. performed experiments. V.S. performed the fabrication of the devices. M.K. supervised the mass spectrometry experiments. W.W.M.P.P. and J.C.d.G. conceptualized and supervised the work. All authors contributed to data analysis, discussion, and interpretation. A.I., E.v.d.W., S.L.M.in't.G., V.S., P.S., D.P., M.K., J.C.d.G., and W.W.M.P.P. wrote the manuscript with input from all the authors.

DECLARATION OF INTERESTS

A.I., E.W., V.S., W.P., and J.G. are inventors on a patent in the field of muscle on a chip.

Received: March 21, 2022
Revised: August 30, 2023
Accepted: August 31, 2023
Published: September 28, 2023

REFERENCES

Afshar, M.E., Abraha, H.Y., Bakooshli, M.A., Davoudi, S., Thavandiran, N., Tung, K., Ahn, H., Ginsberg, H.J., Zandstra, P.W., and Gilbert, P.M. (2020). A 96-well culture platform enables longitudinal analyses of engineered human skeletal muscle microtissue strength. Sci. Rep. *10*, 6918. https://doi.org/10.1038/s41598-020-62837-8.

Afshar Bakooshli, M., Lippmann, E.S., Mulcahy, B., Iyer, N., Nguyen, C.T., Tung, K., Stewart, B.A., van den Dorpel, H., Fuehrmann, T., Shoichet, M., et al. (2019). A 3D culture model of innervated human skeletal muscle enables studies of the adult neuromuscular junction. Elife *8*. https://doi.org/10.7554/eLife.44530.

Benarroch, L., Bonne, G., Rivier, F., and Hamroun, D. (2019). The 2020 version of the gene table of neuromuscular disorders (nuclear genome). Neuromuscul. Disord. *29*, 980–1018. https://doi.org/10.1016/j.nmd.2019.10.010.

Bigot, A., Jacquemin, V., Debacq-Chainiaux, F., Butler-Browne, G.S., Toussaint, O., Furling, D., and Mouly, V. (2008). Replicative aging down-regulates the myogenic regulatory factors in human myoblasts. Biol. Cell. *100*, 189–199. https://doi.org/10.1042/bc20070085.

Borchin, B., Chen, J., and Barberi, T. (2013). Derivation and FACS-mediated purification of PAX3+/PAX7+ skeletal muscle precursors from human pluripotent stem cells. Stem Cell Rep. *1*, 620–631. https://doi.org/10.1016/j.stemcr.2013.10.007.

Buijsen, R.A.M., Gardiner, S.L., Bouma, M.J., van der Graaf, L.M., Boogaard, M.W., Pepers, B.A., Eussen, B., de Klein, A., Freund, C., and van Roon-Mom, W.M.C. (2018). Generation of 3 spinocerebellar ataxia type 1 (SCA1) patient-derived induced pluripotent stem cell lines LUMCi002-A, B, and C and 2 unaffected sibling control induced pluripotent stem cell lines LUMCi003-A and B. Stem Cell Res. *29*, 125–128. https://doi.org/10.1016/j.scr.2018.03.018.

Carvalho, A.A.d.S. (2020). Side Effects of Chloroquine and Hydroxychloroquine on Skeletal Muscle: a Narrative Review. Curr. Pharmacol. Rep. *6*, 364–372. https://doi.org/10.1007/s40495-020-00243-4.

Chal, J., Al Tanoury, Z., Hestin, M., Gobert, B., Aivio, S., Hick, A., Cherrier, T., Nesmith, A.P., Parker, K.K., and Pourquié, O. (2016). Generation of human muscle fibers and satellite-like cells from human pluripotent stem cells *in vitro*. Nat. Protoc. *11*, 1833–1850. https://doi.org/10.1038/nprot.2016.110.

Cho, M., Webster, S.G., and Blau, H.M. (1993). Evidence for myoblast-extrinsic regulation of slow myosin heavy chain expression during muscle fiber formation in embryonic development. J. Cell Biol. *121*, 795–810.

Darabi, R., Arpke, R.W., Irion, S., Dimos, J.T., Grskovic, M., Kyba, M., and Perlingeiro, R.C.R. (2012). Human ES- and iPS-derived myogenic progenitors restore DYSTROPHIN and improve contractility upon transplantation in dystrophic mice. Cell Stem Cell *10*, 610–619. https://doi.org/10.1016/j.stem.2012.02.015.

Day, K., Shefer, G., Shearer, A., and Yablonka-Reuveni, Z. (2010). The depletion of skeletal muscle satellite cells with age is concomitant with reduced capacity of single progenitors to produce reserve progeny. Dev. Biol. *340*, 330–343. https://doi.org/10.1016/j.ydbio.2010.01.006.

DiMasi, J.A., Grabowski, H.G., and Hansen, R.W. (2016). Innovation in the pharmaceutical industry: New estimates of R&D costs. J. Health Econ. *47*, 20–33. https://doi.org/10.1016/j.jhealeco.2016.01.012.

Ebrahimi, M., Lad, H., Fusto, A., Tiper, Y., Datye, A., Nguyen, C.T., Jacques, E., Moyle, L.A., Nguyen, T., Musgrave, B., et al. (2021). De novo revertant fiber formation and therapy testing in a 3D culture model of Duchenne muscular dystrophy skeletal muscle. Acta Biomater. *132*, 227–244. https://doi.org/10.1016/j.actbio.2021.05.020.

Esser, K., Gunning, P., and Hardeman, E. (1993). Nerve-Dependent and -Independent Patterns of mRNA Expression in Regenerating Skeletal Muscle. Dev. Biol. *159*, 173–183. https://doi.org/10.1006/dbio.1993.1231.

Hansen, A., Eder, A., Bönstrup, M., Flato, M., Mewe, M., Schaaf, S., Aksehirlioglu, B., Schwörer, A., Uebeler, J., and Eschenhagen, T. (2010). Development of a Drug Screening Platform Based on Engineered Heart Tissue. Circ. Res. *107*, 35–44. https://doi.org/10.1161/CIRCRESAHA.109.211458.

Iuliano, A., van der Wal, E., Ruijmbeek, C.W.B., in 't Groen, S.L.M., Pijnappel, W.W.M.P., de Greef, J.C., and Saggiomo, V. (2020). Coupling 3D Printing and Novel Replica Molding for In House Fabrication of Skeletal Muscle Tissue Engineering Devices. Adv. Mater. Technol. *5*, 2000344. https://doi.org/10.1002/admt.202000344.

Jiwlawat, S., Lynch, E., Glaser, J., Smit-Oistad, I., Jeffrey, J., Van Dyke, J.M., and Suzuki, M. (2017). Differentiation and sarcomere formation in skeletal myocytes directly prepared from human induced pluripotent stem cells using a sphere-based culture. Differentiation *96*, 70–81. https://doi.org/10.1016/j.diff.2017.07.004.

Kaur, P., Ghariwala, V., Yeo, K.S., Tan, H.Z., Tan, J.C.S., Armugam, A., Strong, P.N., and Jeyaseelan, K. (2012). Biochemistry of Envenomation. In Advances in Clinical Chemistry (Academic Press Inc.), pp. 187–252.

Khodabukus, A. (2021). Tissue-Engineered Skeletal Muscle Models to Study Muscle Function, Plasticity, and Disease. Front. Physiol. *12*, 619710. https://doi.org/10.3389/fphys.2021.619710.

Khodabukus, A., Madden, L., Prabhu, N.K., Koves, T.R., Jackman, C.P., Muoio, D.M., and Bursac, N. (2019). Electrical stimulation increases hypertrophy and metabolic flux in tissue-engineered human skeletal muscle. Organoids Ex Vivo Tissue -Chip Technol *198*, 259–269. https://doi.org/10.1016/j.biomaterials.2018.08.058.

Krivickas, L.S., Dorer, D.J., Ochala, J., and Frontera, W.R. (2011). Relationship between force and size in human single muscle fibres. Exp. Physiol. *96*, 539–547. https://doi.org/10.1113/expphysiol.2010.055269.

Legant, W.R., Pathak, A., Yang, M.T., Deshpande, V.S., McMeeking, R.M., and Chen, C.S. (2009). Microfabricated tissue gauges to measure and manipulate forces from 3D microtissues. Proc. Natl. Acad. Sci. USA *106*, 10097–10102. https://doi.org/10.1073/pnas.0900174106.

Liu, J., Hilderink, J., Groothuis, T.A.M., Otto, C., van Blitterswijk, C.A., and de Boer, J. (2015). Monitoring nutrient transport in tissue-engineered grafts. J. Tissue Eng. Regen. Med. *9*, 952–960. https://doi.org/10.1002/term.1654.

Madden, L., Juhas, M., Kraus, W.E., Truskey, G.A., and Bursac, N. (2015). Bioengineered human myobundles mimic clinical responses of skeletal muscle to drugs. Elife *4*, e04885. https://doi.org/10.7554/eLife.04885.

Maffioletti, S.M., Sarcar, S., Henderson, A.B.H., Mannhardt, I., Pinton, L., Moyle, L.A., Steele-Stallard, H., Cappellari, O., Wells, K.E., Ferrari, G., et al. (2018). Three-Dimensional Human iPSC-Derived Artificial Skeletal Muscles Model Muscular Dystrophies and Enable Multilineage Tissue Engineering. Cell Rep. *23*, 899–908. https://doi.org/10.1016/j.celrep.2018.03.091.

Mills, R.J., Parker, B.L., Monnot, P., Needham, E.J., Vivien, C.J., Ferguson, C., Parton, R.G., James, D.E., Porrello, E.R., and Hudson, J.E. (2019). Development of a human skeletal micro muscle platform with pacing capabilities. Biomaterials *198*, 217–227. https://doi.org/10.1016/j.biomaterials.2018.11.030.

Mueller, C., Trujillo-Miranda, M., Maier, M., Heath, D.E., O'Connor, A.J., and Salehi, S. (2021). Effects of External Stimulators on Engineered Skeletal Muscle Tissue Maturation. Adv. Mater. Interfaces *8*, 2001167. https://doi.org/10.1002/admi.202001167.

Neyroud, D., Cheng, A.J., Donnelly, C., Bourdillon, N., Gassner, A.L., Geiser, L., Rudaz, S., Kayser, B., Westerblad, H., and Place, N. (2019). Toxic doses of caffeine are needed to increase skeletal muscle contractility. Am. J. Physiol. Cell Physiol. *316*. C246-c251. https://doi.org/10.1152/ajpcell.00269.2018.

Osaki, T., Uzel, S.G.M., and Kamm, R.D. (2018). Microphysiological 3D model of amyotrophic lateral sclerosis (ALS) from human iPS-derived muscle cells and optogenetic motor neurons. Sci. Adv. *4*, eaat5847. https://doi.org/10.1126/sciadv.aat5847.

Perez-Riverol, Y., Bai, J., Bandla, C., García-Seisdedos, D., Hewapathirana, S., Kamatchinathan, S., Kundu, D., Prakash, A., Frericks-Zipper, A., Eisenacher, M., et al. (2022). The PRIDE database resources in 2022: A Hub for mass spectrometry-based proteomics evidences. Nucleic Acids Res *50*, D543–D552.

Racca, A.W., Beck, A.E., Rao, V.S., Flint, G.V., Lundy, S.D., Born, D.E., Bamshad, M.J., and Regnier, M. (2013). Contractility and kinetics of human fetal and human adult skeletal muscle. J. Physiol. *591*, 3049–3061. https://doi.org/10.1113/jphysiol.2013.252650.

Rao, L., Qian, Y., Khodabukus, A., Ribar, T., and Bursac, N. (2018). Engineering human pluripotent stem cells into a functional skeletal muscle tissue. Nat. Commun. *9*, 126. https://doi.org/10.1038/s41467-017-02636-4.

Romero, N.B., Mezmezian, M., and Fidzińska, A. (2013). Main steps of skeletal muscle development in the human: Morphological analysis and ultrastructural characteristics of developing human muscle. Handb. Clin. Neurol. *113*, 1299–1310. https://doi.org/10.1016/B978-0-444-59565-2.00002-2.

Sakar, M.S., Neal, D., Boudou, T., Borochin, M.A., Li, Y., Weiss, R., Kamm, R.D., Chen, C.S., and Asada, H.H. (2012). Formation and optogenetic control of engineered 3D skeletal muscle bioactuators. Lab Chip *12*, 4976–4985. https://doi.org/10.1039/C2LC40338B.

Schiaffino, S., Rossi, A.C., Smerdu, V., Leinwand, L.A., and Reggiani, C. (2015). Developmental myosins: expression patterns and functional significance. Skeletal Muscle *5*, 22. https://doi.org/10.1186/s13395-015-0046-6.

Selvaraj, S., Mondragon-Gonzalez, R., Xu, B., Magli, A., Kim, H., Lainé, J., Kiley, J., Mckee, H., Rinaldi, F., Aho, J., et al. (2019). Screening identifies small molecules that enhance the maturation of human pluripotent stem cell-derived myotubes. Elife *8*, e47970. https://doi.org/10.7554/eLife.47970.

Takahashi, K., and Yamanaka, S. (2016). A decade of transcription factor-mediated reprogramming to pluripotency. Nat. Rev. Mol. Cell Biol. *17*, 183–193. https://doi.org/10.1038/nrm.2016.8.

Vandenburgh, H., Shansky, J., Benesch-Lee, F., Barbata, V., Reid, J., Thorrez, L., Valentini, R., and Crawford, G. (2008). Drug-screening platform based on the contractility of tissue-engineered muscle. Muscle Nerve *37*, 438–447. https://doi.org/10.1002/mus.20931.

van der Wal, E., Bergsma, A.J., van Gestel, T.J.M., in 't Groen, S.L.M., Zaehres, H., Araúzo-Bravo, M.J., Schöler, H.R., van der Ploeg, A.T., and Pijnappel, W.W.M.P. (2017). GAA Deficiency in Pompe Disease Is Alleviated by Exon Inclusion in iPSC-Derived Skeletal Muscle Cells. Mol. Ther. Nucleic Acids *7*, 101–115. https://doi.org/10.1016/j.omtn.2017.03.002.

van der Wal, E., Herrero-Hernandez, P., Wan, R., Broeders, M., Pijnappel, W.W.M.P., In 't Groen, S.L.M., van Gestel, T.J.M., van IJcken, W.F.J., Cheung, T.H., van der Ploeg, A.T., and Schaaf, G.J. (2018). Large-Scale Expansion of Human iPSC-Derived Skeletal Muscle Cells for Disease Modeling and Cell-Based Therapeutic Strategies. Stem Cell Rep. *10*, 1975–1990. https://doi.org/10.1016/j.stemcr.2018.04.002.

van der Wal, E., den Hamer, B., van der Vliet, P.J., Tok, M., Brands, T., Eussen, B., Lemmers, R.J.L.F., Freund, C., de Klein, A., Buijsen, R.A.M., et al. (2019). Generation of genetically matched hiPSC lines from two mosaic facioscapulohumeral dystrophy type 1 patients. Stem Cell Res. *40*, 101560. https://doi.org/10.1016/j.scr.2019.101560.

Wang, J., Zhou, C.J., Khodabukus, A., Tran, S., Han, S.O., Carlson, A.L., Madden, L., Kishnani, P.S., Koeberl, D.D., and Bursac, N. (2021). Three-dimensional tissue-engineered human skeletal muscle model of Pompe disease. Commun. Biol. *4*, 524. https://doi.org/10.1038/s42003-021-02059-4.

Wehrle, U., Düsterhöft, S., and Pette, D. (1994). Effects of chronic electrical stimulation on myosin heavy chain expression in satellite cell cultures derived from rat muscles of different fiber-type composition. Differentiation *58*, 37–46. https://doi.org/10.1046/j.1432-0436.1994.5810037.x.

Xu, B., Zhang, M., Perlingeiro, R.C.R., and Shen, W. (2019). Skeletal Muscle Constructs Engineered from Human Embryonic Stem Cell Derived Myogenic Progenitors Exhibit Enhanced Contractile Forces When Differentiated in a Medium Containing EGM-2 Supplements. Adv. Biosyst. *3*, e1900005.

Zhou, J., Parker, D.C., White, J.P., Lim, A., Huffman, K.M., Ho, J.P., Yen, P.M., and Kraus, W.E. (2019). Thyroid Hormone Status Regulates Skeletal Muscle Response to Chronic Motor Nerve Stimulation. Front. Physiol. *10*, 1363. https://doi.org/10.3389/fphys.2019.01363.

Stem Cell Reports, Volume *18*

Supplemental Information

Highly contractile 3D tissue engineered skeletal muscles from human iPSCs reveal similarities with primary myoblast-derived tissues

Erik van der Wal, Alessandro Iuliano, Stijn L.M. in 't Groen, Anjali P. Bholasing, Dominik Priesmann, Preeti Sharma, Bianca den Hamer, Vittorio Saggiomo, Marcus Krüger, W.W.M. Pim Pijnappel, and Jessica C. de Greef

Supplemental figures

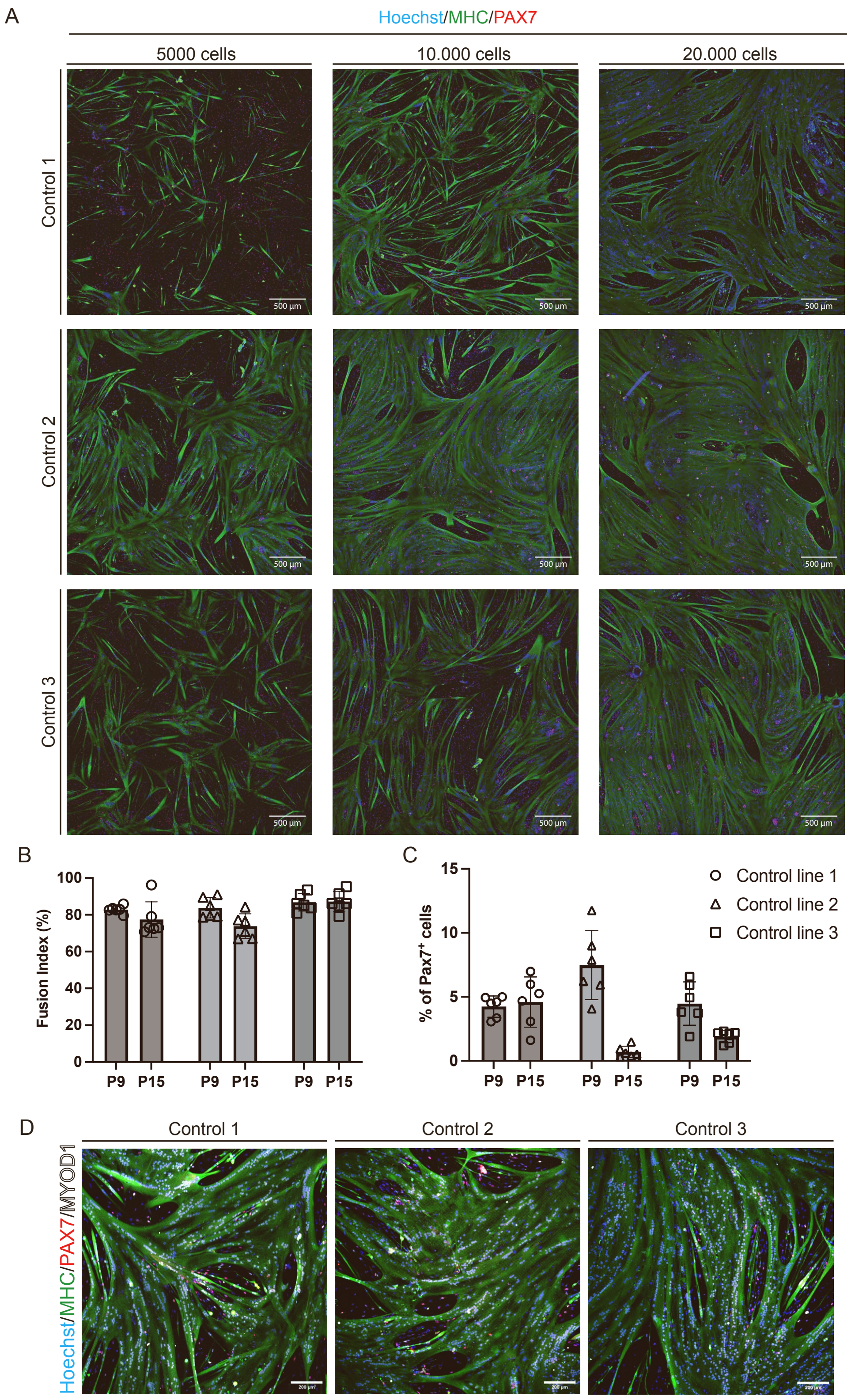

Figure S1 (related to Figure 1). Control 1-3 myogenic progenitors. (A) Staining of MPs of control 1-3 differentiated for 4 days with MYH (green) and PAX7 (red) at different cell densities. Nuclei were

stained with Hoechst (blue). (B) Quantification of fusion index for control 1-3 at passage 9 (P9) and passage 15 (P15) of culture. C) Quantification of percentage of Pax7$^+$ cells in the culture of differentiated MPs of control 1-3 at P9 and P15. D) Immunostaining of MPs from control lines 1-3 differentiated for 4 days with MYH (green), PAX7 (red), MYOD1 (white) and nuclear staining with Hoechst (blue).

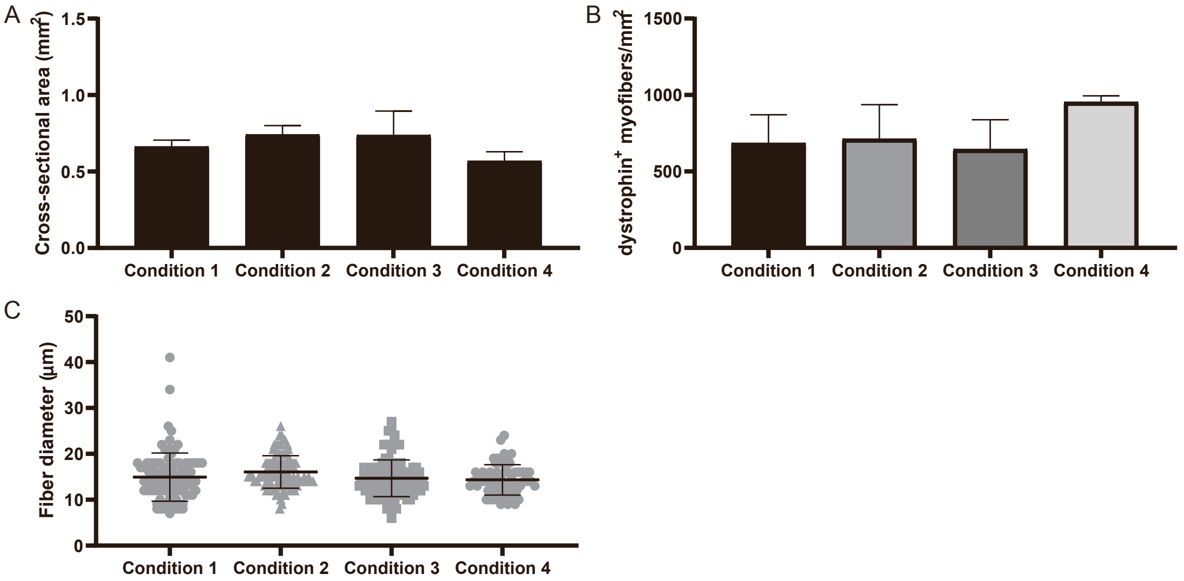

Figure S2 (related to Figure 2). Cross-sectional area, number of dystrophin$^+$ myofibers and myofiber diameter. A) 3D-TESMs were differentiated in conditions 1-4 and on day 7 of differentiation the cross-sectional area was determined. B) Same as (A), but with the number of dystrophin$^+$ myofibers corrected for the cross-sectional area (see Figure 2C). C) Same as (B), but then for myofiber diameter. Five random fields were selected per cross-section and at least 100 myofibers were measured. Data is derived from three independent 3D-TESMs and expressed as mean ± SD.

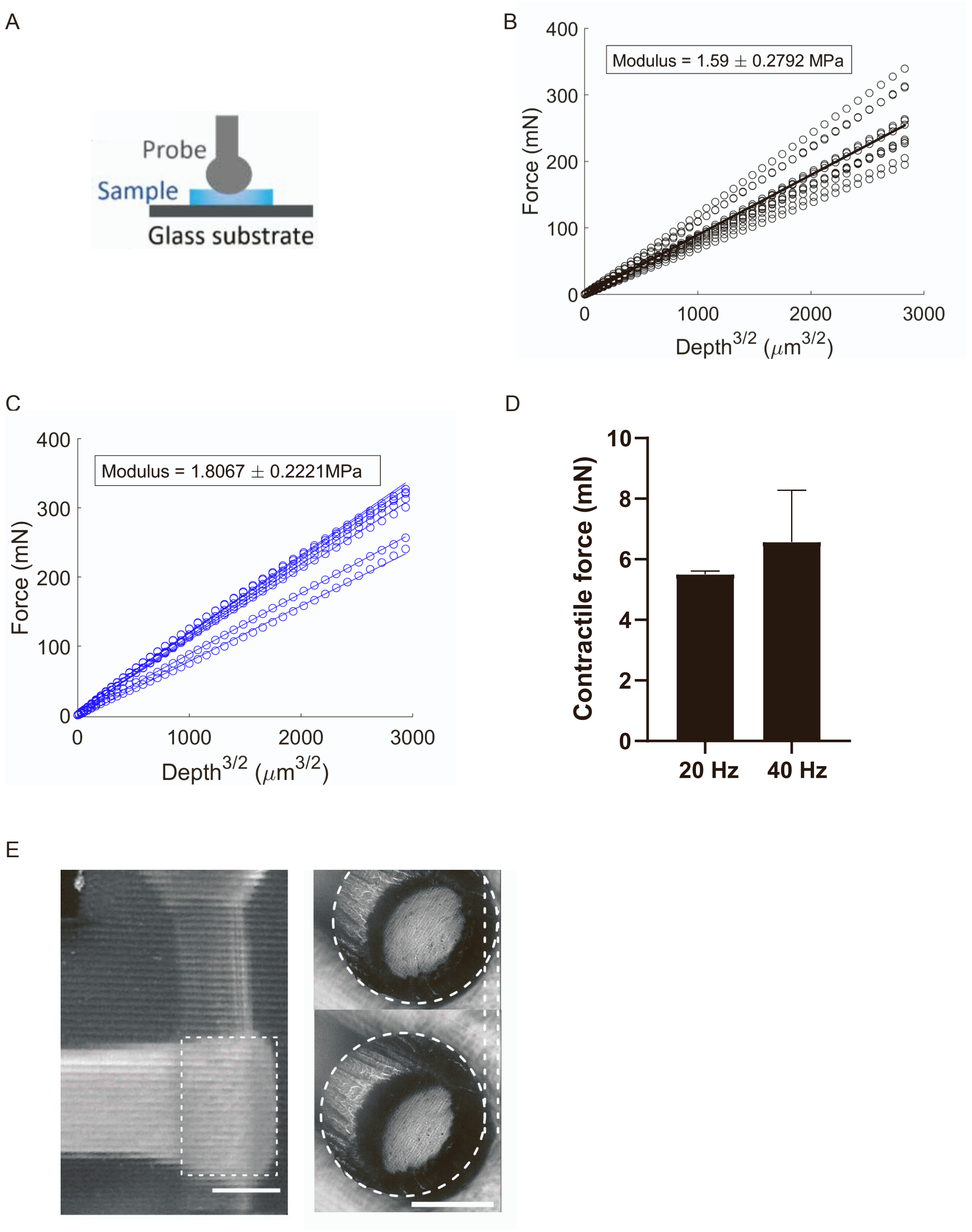

Figure S3 (related to Figures 3 and 5). PDMS stiffness and absolute contractile force. A) PDMS stiffness was measured using indentation method. B) Measured Young's Modulus for the Direct Peeling platform from 15 independent PDMS chambers. C) Measured Young's Modulus of Ecoflex Replica platform from 7 independent PDMS chambers. Plotted line indicates average of all samples. D) Absolute contractile force of 3D-TESMs from control 1 myogenic progenitors stimulated with 20 Hz and 40 Hz on day 7 of differentiation from three independent 3D-TESMs Data is expressed as mean ± SD. E) Position of 3D-TESMs on the pillar of an Ecoflex chamber (left) and top view of pillar displacement (right) before and during stimulation.

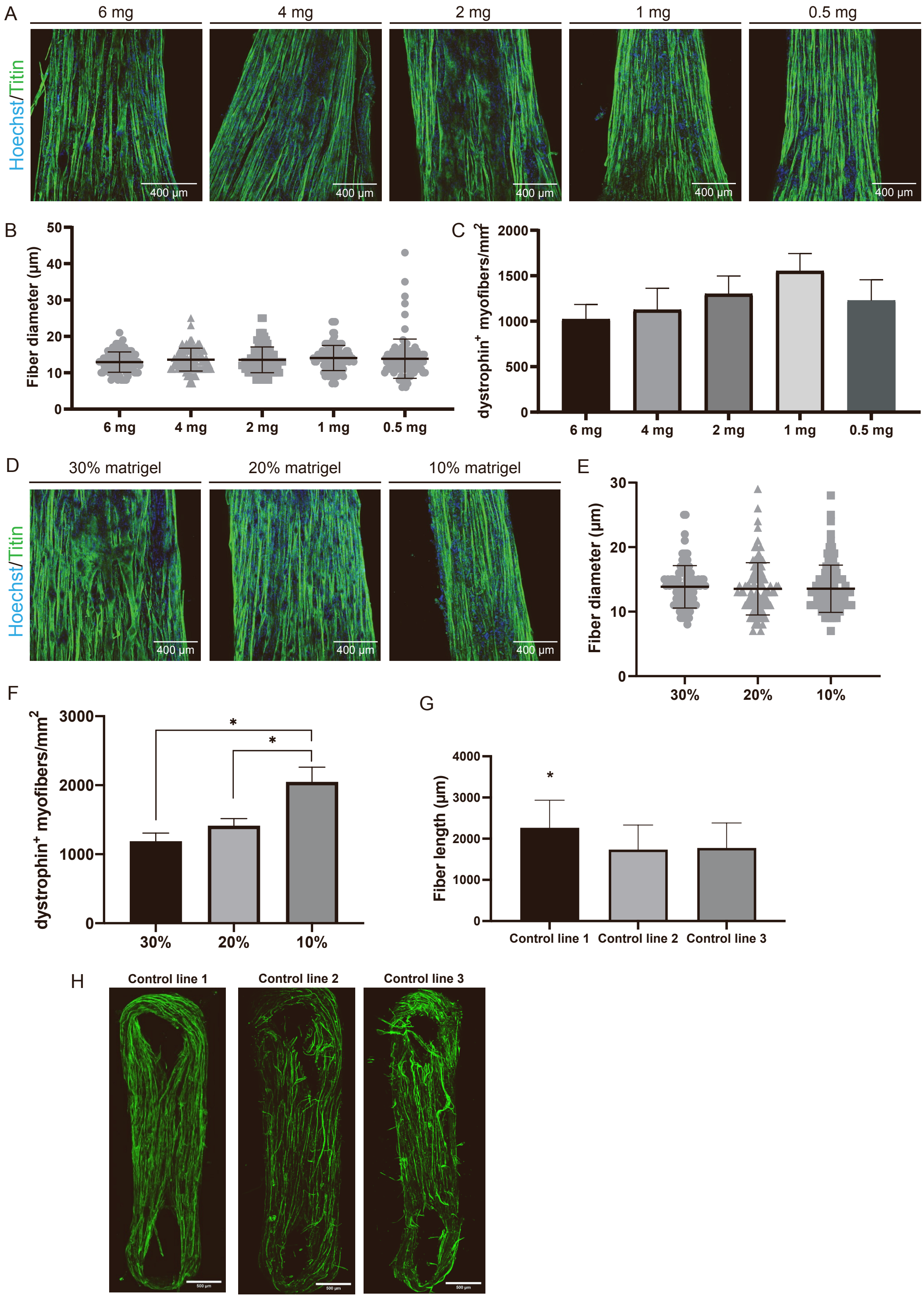

Figure S4 (related to Figure 4). Varying fibrinogen and Matrigel concentrations in the hydrogel of 3D-TESMs. A) 3D-TESMs stained for titin (green) and Hoechst (blue) using whole mount staining on day 7 of differentiation. B) Average myofiber diameter ± SD measured from 3 random fields on 3 cross-sections per fibrinogen concentration derived from Figure 4A. C) Same as (B) but for the average

number of dystrophin$^+$ myofibers. D) Same as (A) but for different Matrigel concentrations. E) Same as (B) showing myofiber diameter quantified from cross-sections of Figure 4E. F) Same as (E) but for the average number of dystrophin$^+$ myofibers**.** Data is derived from three independent 3D-TESMs and expressed as mean ± SD. G) Quantification of fiber length (n=20) in 3D-TESMs generated with the Ecoflex Replica platform from the three control lines. H) Whole-tissue immunostaining for titin (green) of representative tissues generated in the Ecoflex Replica platform for control lines 1-3.

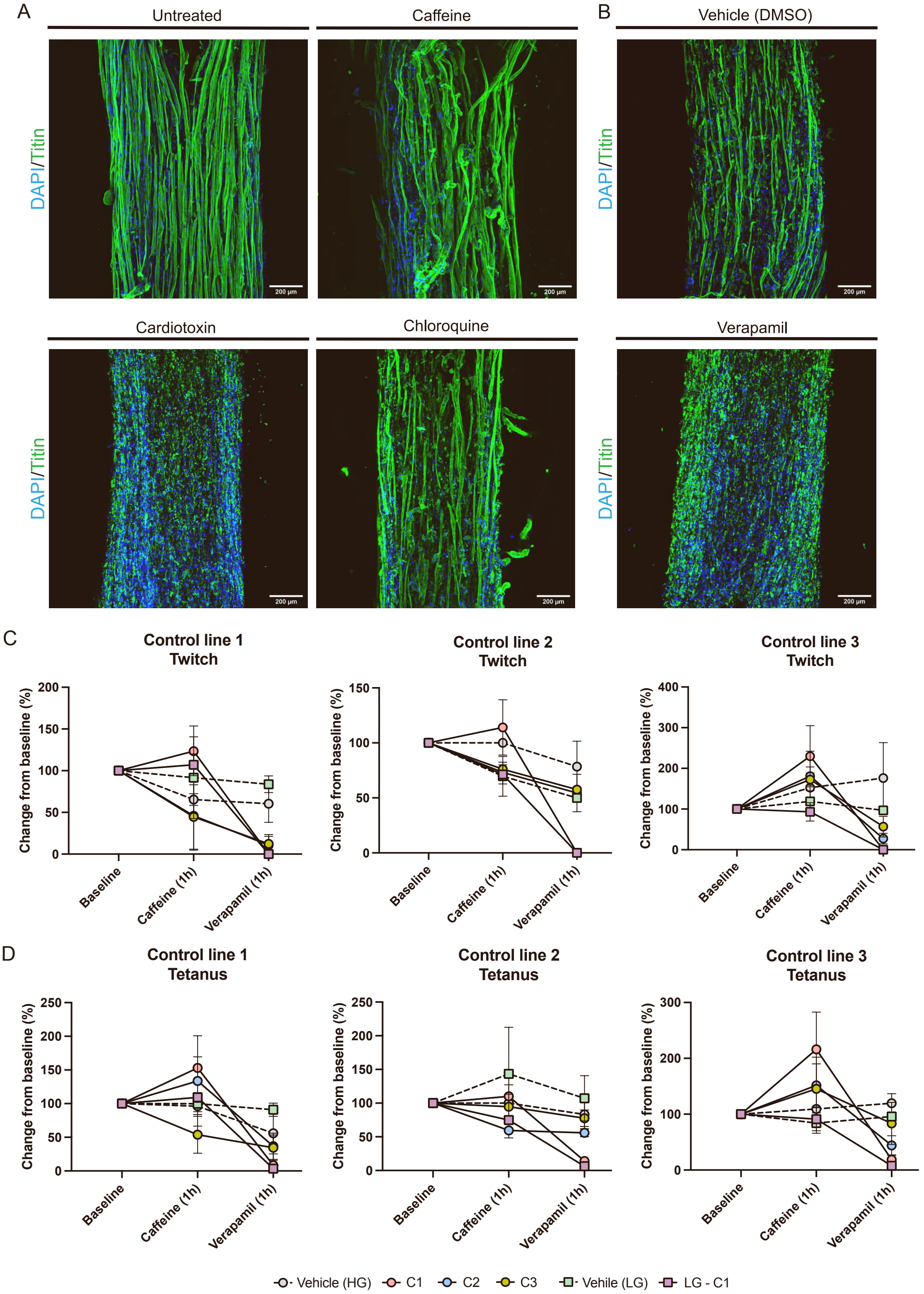

Figure S5 (related to Figure 5). Drug testing on 3D-TESMs from Ecoflex Replica platform. A) Whole mount staining of 3D-TESMs treated with drugs (caffeine, cardiotoxin, chloroquine) for 6 hours using an antibody against titin (green) and nuclei stained with DAPI (blue). C) Same as (D) but then for verapamil, incubated for 1 hour. C) Quantification of change in absolute twitch force after 1h of

administration of caffeine and verapamil, for control lines 1-3. Dashed lines identify vehicle controls. D) Same as (C), but for absolute tetanic force.

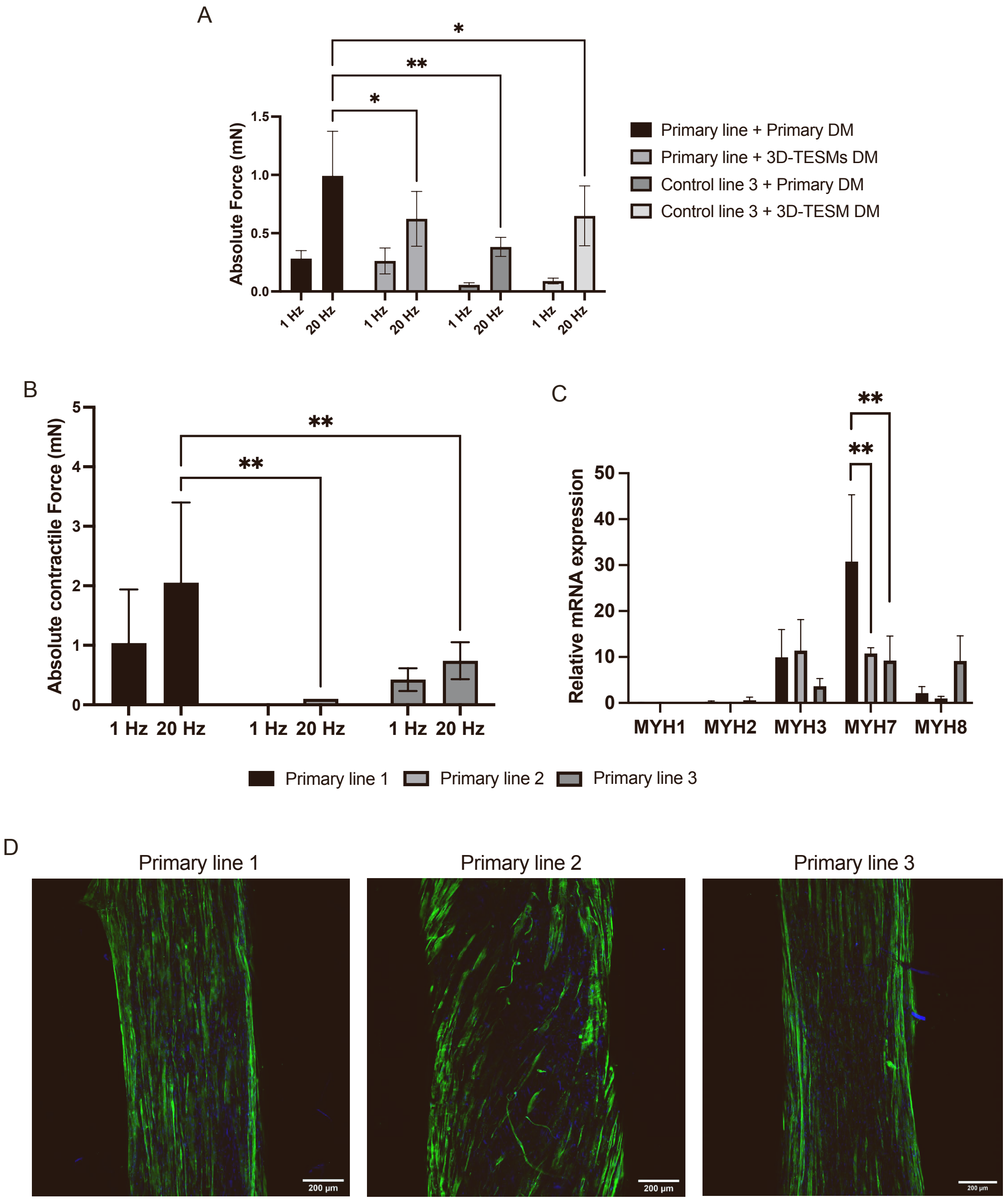

Figure S6 (related to Figure 6). Comparison of three independent primary myoblast lines. A) Comparison of contractile force generated by primary myoblasts derived tissues and myogenic progenitors-derived 3D-TESMs under different media formulations. B) Absolute force quantification for primary lines 1, 2 and 3 at D7 of differentiation of 3D-TESMs generated from the Ecoflex Replica platform. C) RT-qPCR gene expression analysis for the three primary lines. D) Whole-mount immunostaining of representative tissues of the three primary lines. Important disclaimer: any difference in the gene expression data relative to the primary lines compared to the main manuscript is due to intrinsic operator-dependent variability.

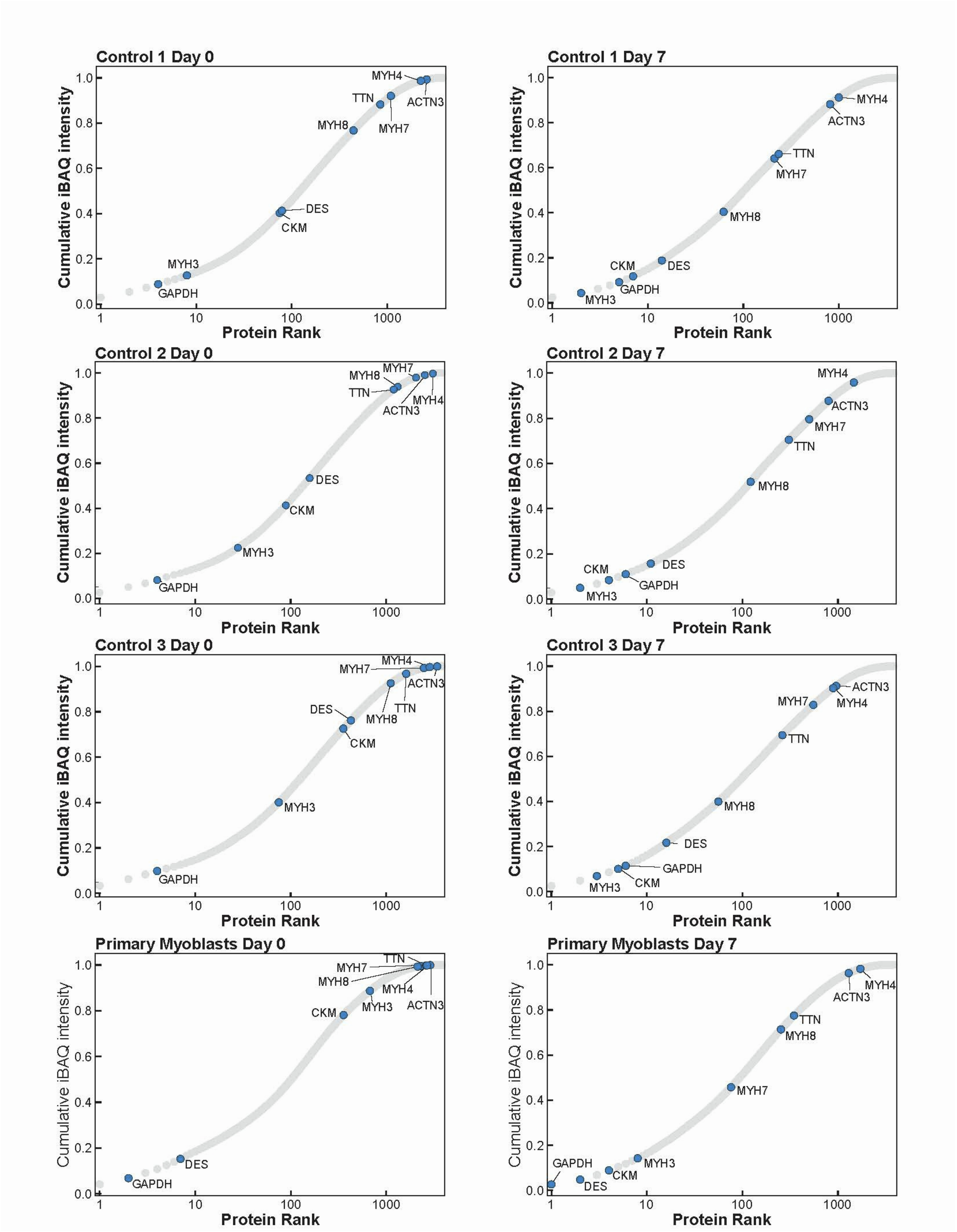

Figure S7 (related to Figure 7). Protein rankings in hiPSC-derived and primary myoblast-derived 3D-TESMs. Protein ranking overview at day 0 (left) and day 7 (right) of differentiation for 3D-TESMs derived from controls 1-3 and primary myoblasts. Protein ranking is based on iBAQ of individual proteins on the x-axis; whereby rank 1 represents the most abundant protein in the sample, while the cumulative iBAQ intensity is represented on the y-axis. Data is derived from three independent 3D-TESMs and expressed as mean.

Supplemental methods

Fabrication of Direct Peeling and Ecoflex Replica-based PDMS chambers

PDMS chambers for the Direct Peeling platform (50 µl) or Ecoflex Replica platform (15 µl) were fabricated as previously described (Iuliano et al., 2020). Briefly, for the Direct Peeling platform, negative molds were printed with an Ultimaker 2$^+$ (Ultimaker) FDM printer with ABS material (Ultimaker). SYLGARD™ 184 (PDMS) Base and Curing agent (Dow Corning) were thoroughly mixed in a 10:1 ratio, degassed under vacuum, poured on top of the negative Direct Peeling mold and incubated for 2 hours at 75 °C. For the Ecoflex Replica platform, a two-step replica molding methodology was performed. Positive master molds were generated with SLA 3D printing (Form 3, Formlabs) and negative molds were generated of Ecoflex-00-30 material (Smooth-On Inc). PDMS chambers were released after casting by stretching of the Ecoflex-00-30 negative mold. Prior to generation of 3D-TESMs, PDMS chambers were pretreated with 1% Pluronic F127 (Sigma-Aldrich) for at least 1 hour at room temperature.

Human primary myoblast culture

Human primary myoblasts (Rf745.1) received from the University of Rochester biorepository (https://www.urmc.rochester.edu/neurology/fshd-center/research-info/sharingbiologicalresources.aspx) (Balog et al., 2012) were cultured using previously defined conditions (Madden et al., 2015). In short, during expansion myoblasts were cultured on ECM-coated dishes in DMEM low glucose supplemented with SkGM bullet kit (Lonza: CC-3245) excluding insulin (GM primary). For 3D-TESM formation of human primary myoblasts, the same hydrogel composition and number of cells were used as for 3D-TESMs generated with myogenic progenitors. After formation, 3D-TESMs were first cultured in GM primary medium supplemented with 1.5 mg/ml 6-ACA and after 2 days differentiation was induced with DM primary medium (DMEM low glucose supplemented with 2% horse serum (Gibco), 2 mg/ml 6-ACA and 10 µg/ml insulin).

RNA isolation

Prior to RNA isolation, 3D-TESMs were washed once with PBS, detached from pillars, snap-frozen in liquid nitrogen and stored at -80 °C. For RNA isolation 3D-TESMs were dissolved in 700 µl QIAzol lysis buffer (Qiagen) and disrupted with a tissueRuptor (Qiagen) for 5 seconds. After disruption, RNA was isolated with the mRNeasy mini kit or RNeasy micro kit (Qiagen) including the DNase step, all according to the manufacturer's manual.

cDNA synthesis and RT-qPCR

Equal amounts of RNA were used for cDNA synthesis using Fermentas cDNA synthesis kit (Fermentas) according to the manufacturer's manual. For RT-qPCR, cDNA was either 5× or

10× diluted and performed as described previously (van der Wal et al., 2017). Primers are shown in Table S1.

Whole mount immunofluorescence staining

3D-TESMs were washed once with PBS, fixed with 4% paraformaldehyde (PFA; Sigma-Aldrich) for 1 hour at room temperature and stored in PBS at 4 °C. For staining, 3D-TESMS were blocked for 1 hour in blocking buffer containing 3% BSA, 0.1% Tween-20 and 0.3% Triton X-100 (all Sigma-Aldrich). Primary and secondary antibodies (Table S1) were diluted in antibody dilution buffer (0.1% BSA, 0.1% Tween-20, 0.3% Triton-X100). Primary antibodies were incubated for 1 hour at room temperature while shaking (150 rpm), followed by washing with PBS-0.1% Tween-20 and with PBS. Thereafter, 3D-TESMs were incubated for 30 minutes at room temperature with secondary antibodies combined with Hoechst nuclear staining. 3D-TESMs were washed once with PBS-0.1% Tween-20 and imaged using an Andor Dragonfly 200 or Leica TCS SP5 Confocal microscope.

Indentation method to measure Young's Modulus of PDMS chambers

Young's modulus of the samples was measured with a custom-made indentation set-up. A spherical glass probe with a diameter of 2 mm was used to indent PDMS sample, which consisted of a flat piece cut from the chamber and was fixed onto a glass substrate via plasma bonding. Step size was 5 µm with an indentation depth of 200 µm. The elastic response was measured via a load cell (250 gm, Futek) with a sampling rate of 1000. An average of 1000 points was plotted versus the indentation depth. Multiple experiments were performed on each sample with varying locations. Young's Modulus (Y) was determined by fitting the indentation depth (*d*), as a function of measured indenter load (F) during approach via following equation (Harding and Sneddon, 1945):

$$F = \frac{4}{3}\frac{Y}{1-\nu^2}d^{3/2}R^{1/2}$$

R is the radius of the indenting sphere (1 mm) and n is the Poisson ratio (0.5). The slope of the linear fit allowed us to extract Young's modulus (Y).

Drug treatment of 3D-TESMs

For drug treatments, 3D-TESMs were incubated with either caffeine (C0750-100G, Sigma-Aldrich), chloroquine (C6628-25G, Sigma-Aldrich-) or cardiotoxin from *Naja pallida* (L8102, Latoxan), all dissolved in water. Verapamil (V4629-1G, Sigma-Aldrich) was dissolved in DMSO as per manufacturer instructions, DMSO was included as a vehicle treatment at a final concentration of 0.1 % v/v. All drugs were diluted in differentiation medium. 3D-TESMs treated with caffeine, chloroquine and cardiotoxin were incubated for 6 hours before analysis. 3D-TESMs treated with verapamil and vehicle were incubated for 1 hour before analysis.

Proteomic analysis

3D-TESMs generated in Ecoflex Replica-based chambers were used for proteomic analysis. For analyses, three 3D-TESMs per biological replica were pooled. Three biological replicas were used per cell line and differentiated for 0 or 7 days. After culturing, all 3D-TESMs were immediately snap-frozen in liquid nitrogen and stored at -80 °C. As a negative control, three cell-free tissues were included containing only fibrinogen, thrombin and Matrigel and were snap-frozen after 30 minutes of polymerization. Tissues were resuspended in 50 µL urea buffer (6 M urea, 2 M thiourea in 10 mM HEPES/KOH pH 8.0) and sonicated for complete disruption. Proteins were reduced and alkylated with 5 mM TCEP and 15 mM CAA at room temperature for 30 minutes. Samples were digested with endoproteinase Lys-C (Wako) at an enzyme_substrate ratio of 1:100 for 3 hours, diluted with three sample volumes of 50 mM ammonium bicarbonate (ABC) buffer and further digested with Trypsin (Sigma Aldrich) at an enzyme_substrate ratio of 1:100 at room temperature overnight. Proteases were inactivated by adding formic acid to 1% and undigested proteins were removed by centrifugation (20,000 x g, RT, 10 min). Peptides were desalted on SDB-RPS StageTips.

Proteomics data were acquired on an Orbitrap Eclipse Tribrid mass spectrometer equipped with a FAIMS PRO device, coupled to an EASY-nLC 1200 UHPLC system (Thermo Scientific) equipped with a 50 cm column packed in-house with C18 *Poroshell* beads (2.7 µm, Agilent). A two-hour gradient within a binary solvent system consisting of 0.1% formic acid (buffer A) and 0.1% formic acid in 80% acetonitrile (buffer B) was used. Buffer B was increased from 4% to 35% within 98 minutes and further to 55% within 12 minutes. The gradient was ramped up to 95% buffer B within 4 minutes and maintained for 6 minutes to elute any remaining peptides. Full MS scans from 350-1400 m/z were acquired at a resolution of 60,000, an AGC target of 1.2E7 and a maximum injection time of 50 ms. Fragmentation scans were acquired at a resolution of 15,000, an AGC target of 1.5E6 and a maximum injection time of 22 ms. A total of 12, 10 and 8 fragmentation scans were acquired at FAIMS compensation voltages of -50 V, -65 V and -80 V, respectively.

Raw files were processed in MaxQuant (v. 1.6.14) using implemented Andromeda search engine (Cox et al., 2011). Peptides and proteins were identified using human Uniprot database (FASTA file downloaded 13/9/2019) with common contaminants. All parameters were set to default values and Trypsin/P was chosen as protease. Methionine oxidation and N-terminal acetylation were set as variable modifications and cysteine carbamidomethylation was considered a fixed modification. The “match between runs” algorithm was enabled, and label-free quantification (LFQ) was performed based on proteotypic peptide intensities.

Statistical analysis was performed in Perseus (v. 1.6.15) (Tyanova et al., 2016). Potential contaminants and reverse peptides were filtered and LFQ intensities were log2-transformed. Data were normalized by column-wise median subtraction and two-sided t-tests were performed to identify differentially expressed proteins. Permutation-based false discovery rate (FDR) estimation was performed with a Fudge factor (S_0) of 0.1 and 500 permutations.

The FDR cutoff was set to 0.05. Identified protein groups were annotated with Gene Ontology terms for Cellular Component, Molecular Function and Biological Process. Graphical visualization was done in Instant Clue.

Tissue sectioning

For the Direct Peeling-based chambers, detached 3D-TESMs were fixed for 1 hour with 4% PFA, washed with PBS and incubated for 2 days in 30% sucrose solution (Sigma-Aldrich) in PBS at 4 °C. Next, 3D-TESMs were embedded in OCT in plastic cryomolds (both Tissue-Tek), snap frozen in liquid nitrogen cooled isopentane (Sigma-Aldrich) and stored at -80 °C. 20 µM thick sections were generated and after cryosectioning slices were stored at -20 °C. For staining, antigen retrieval was performed with Tris/EDTA (10 mM Tris, 1.25 mM EDTA pH9) buffer for 15 minutes while boiling. Staining was performed as described above and after the last step, tissues were dried and mounted with Prolong Diamond antifade mountant (Thermo Fisher Scientific).

Due to the small size of 3D-TESMs generated in Ecoflex Replica-based chambers, either gelatin embedding or paraffin embedding was performed. Gelatin embedding is gentler on tissues, thus it was used to maintain shape and size of engineered tissues for later quantifications. Before embedding, 3D-TESMS were fixed with 4% PFA and then incubated in 10% sucrose dissolved in water overnight at 4 °C. Multiple tissue replicas were then placed on a single block of gelatin containing 30% sucrose and covered with an additional layer of the same solution for a complete embedding. Subsequently, embedded tissue blocks were incubated overnight at 4 °C in a 30% sucrose water-based solution for equilibration and ultimately subjected to a final fixation step in the same solution with addition of 10% PFA ON at 4 °C. Tissue blocks were ultimately freeze-cut in sections of 30 µm thickness using a cryotome. Before mounting on glass slides for imaging, sections were subjected to a free-floating immunostaining procedure identical to whole mount immunostaining used for entire tissues. Paraffin embedding was instead chosen merely for fast qualitative assessments. Fixed tissues were embedded in paraffin blocks through a standard protocol using a histological sample preparation machine (EFTP, Intelsint, Italy) and cut in sections of 6 µm thickness using a microtome. Sections were then mounted on glass slides and subjected to immunostaining.

Image analysis, quantification of cross-sectional area (CSA), myofiber diameter and length

To process the images obtained after immunofluorescence staining the software ImageJ was used. The same software was used to perform quantification of CSA and fiber diameter and length from the images of the 3D-TESMs. Three (n=3) tissues per line and per condition were used for each analysis, cross sections belonging to the central portion of each tissue were chosen for further quantifications. Two different levels of image threshold were chosen to analyze the CSA and the fiber diameter, respectively. The levels of threshold were maintained constant across multiple pictures. To quantify the fiber diameter, we chose only fibers that were both positive for dystrophin immunostaining and showed a complete positive

perimeter. All the candidate fibers visible in each section were selected. In order to minimize the possible bias coming from the irregular shape of the fibers, we semi-randomly selected the axis of each fiber to be chosen as diameter. To quantify the length of the fibers we performed tile scans of whole-mount immunostained tissues. Three tissues per line were chosen and the length of fibers positive for titin was measured. Only fibers whose beginning and end was clearly trackable were chosen and segmented lines were traced along each fiber (n=20 per tissue).

Data and materials availability

All data needed to evaluate conclusions in the paper are present in the paper and/or the supplemental information. Raw/processed data required to reproduce these findings cannot be shared at this time due to technical or time limitations. Additional data related to this paper may be requested from the authors.

Supplemental tables

Table S1. Antibodies and primers used

Name	Dilution or Sequence 5'-3'	Company	Assay
Mouse-anti-MF20	1:50	DSHB	IF
Mouse-anti-PAX7	1:100	DSHD	IF
Mouse-anti-Titin	1:50	DSHB	IF, IHC
Mouse-anti-Brachyury	1:200	R&D (AF2085)	IF
Rabbit-anti-Dystrophin	1:100 or 1:500	Abcam (ab15277)	IHC
MYH1_fw	CTCCTCTTTGTTGGGGCAAC	IDTDNA	RT-qPCR
MYH1_rv	CAGCTTATTCAAATTCTCCC	IDTDNA	RT-qPCR
MYH2_fw	TAAAAAGCTCCAAGAACTGT	IDTDNA	RT-qPCR
MYH2_rv	TGCGCTCCCTTTCAGACTTT	IDTDNA	RT-qPCR
MYH3_fw	CTTGTGGGCGGAGGTCTG	IDTDNA	RT-qPCR
MYH3_rv	AGCAGCTATGCCGAACACTT	IDTDNA	RT-qPCR
MYH4_fw	CTTGAAGTAGTTGTCTGCTTTG	IDTDNA	RT-qPCR
MYH4_rv	GGCTGCAGGTTATTGATGGC	IDTDNA	RT-qPCR
MYH7_fw	CTGTCCAAGTTCCGCAAGGT	IDTDNA	RT-qPCR
MYH7_rv	TCATTCAAGCCCTTCGTGCC	IDTDNA	RT-qPCR
MYH8_fw	ATTTCCACCAAGAACCCA	IDTDNA	RT-qPCR
MYH8_rv	AAAGGATTCTGCCTCTGG	IDTDNA	RT-qPCR

Table S2. Medium composition of condition 1-4

Condition	Composition	References
Condition 1	DMEM High glucose, 1% penicillin, 1% ITS-X, 2 mg/ml 6-ACA	(Borchin et al., 2013)
Condition 2	DMEM Low glucose, 1% penicillin, 2% N2, 2 mg/ml 6-ACA	(Rao et al., 2018)

Condition 3	DMEM Low glucose, 2% horse serum, 10 µg/ml insulin, 1% penicillin, 2 mg/ml 6-ACA	(Afshar et al., 2020; Afshar Bakooshli et al., 2019)
Condition 4	DMEM High glucose, 1% Pen, 1% ITS-X, 1% KOSR, 2 mg/ml 6-ACA	(van der Wal et al., 2018)

Supplemental references

Balog J., Thijssen PE., de Greef JC., Shah B., van Engelen BG., Yokomori K., Tapscott SJ., Tawil R., van der Maarel SM. (2012) Correlation analysis of clinical parameters with epigenetic modifications in the DUX4 promoter in FSHD. Epigenetics. *6*, 579-84.

Cox, J., Neuhauser, N., Michalski, A., Scheltema, R.A., Olsen, J.V., and Mann, M. (2011). Andromeda: A Peptide Search Engine Integrated into the MaxQuant Environment. J. Proteome Res. *10*, 1794–1805.

Harding, J.W., and Sneddon, I.N. (1945). The elastic stresses produced by the indentation of the plane surface of a semi-infinite elastic solid by a rigid punch. Math. Proc. Camb. Philos. Soc. *41*, 16–26.

Iuliano, A., van der Wal, E., Ruijmbeek, C.W.B., in 't Groen, S.L.M., Pijnappel, W.W.M.P., de Greef, J.C., and Saggiomo, V. (2020). Coupling 3D Printing and Novel Replica Molding for In House Fabrication of Skeletal Muscle Tissue Engineering Devices. Adv. Mater. Technol. *5*, 2000344.

Madden, L., Juhas, M., Kraus, W.E., Truskey, G.A., and Bursac, N. (2015). Bioengineered human myobundles mimic clinical responses of skeletal muscle to drugs. ELife.

Tyanova, S., Temu, T., Sinitcyn, P., Carlson, A., Hein, M.Y., Geiger, T., Mann, M., and Cox, J. (2016). The Perseus computational platform for comprehensive analysis of (prote)omics data. Nat. Methods *13*, 731–740.

van der Wal, E., Bergsma, A.J., van Gestel, T.J.M., in 't Groen, S.L.M., Zaehres, H., Araúzo-Bravo, M.J., Schöler, H.R., van der Ploeg, A.T., and Pijnappel, W.W.M.P. (2017). GAA Deficiency in Pompe Disease Is Alleviated by Exon Inclusion in iPSC-Derived Skeletal Muscle Cells. Mol. Ther. - Nucleic Acids *7*, 101–115.
